# MiR-139 Modulates Cancer Stem Cell Function of Human Breast Cancer through Targeting CXCR4

**DOI:** 10.3390/cancers13112582

**Published:** 2021-05-25

**Authors:** Chun-Wen Cheng, Wen-Ling Liao, Po-Ming Chen, Jyh-Cherng Yu, Hui-Ping Shiau, Yi-Hsien Hsieh, Huei-Jane Lee, Yu-Chun Cheng, Pei-Ei Wu, Chen-Yang Shen

**Affiliations:** 1 Institute of Medicine, Chung Shan Medical University, Taichung 40201, Taiwan; A33174@mail.cmuh.org.tw (P.-M.C.); shanchung905@gmail.com (H.-P.S.); hyhsien@csmu.edu.tw (Y.-H.H.); 2 Clinical Laboratory, Chung Shan Medical University Hospital, Taichung 40201, Taiwan; 3 Graduate Institute of Integrated Medicine, China Medical University, Taichung 40433, Taiwan; wl0129@mail.cmu.edu.tw; 4 Center for Personalized Medicine, China Medical University Hospital, Taichung 40433, Taiwan; 5 Department of Surgery, Tri-Service General Hospital, National Defense Medical College, Taipei 11490, Taiwan; doc20106@ndmctsgh.edu.tw; 6 Department of Biochemistry, School of Medicine, Chung Shan Medical University, Taichung 40201, Taiwan; lhj@csmu.edu.tw; 7 School of Medicine, Fu Jen Catholic University, New Taipei 24206, Taiwan; yuchuncheng0817@gmail.com; 8 Institute of Biomedical Sciences, Academia Sinica, Taipei 11529, Taiwan; peiei@gate.sinica.edu.tw; 9 Graduate Institute of Environmental Science, China Medical University, Taichung 40433, Taiwan

**Keywords:** breast cancer, microRNA, CXCR4, cancer stem cell, biomarker

## Abstract

**Simple Summary:**

The C-X-C motif chemokine receptor 4 (CXCR4) is overexpressed in various cancer stem/progenitor cells via activation of the epithelial-mesenchymal transition (EMT) program to facilitate tumor cell aggressiveness in the premetastatic niche. Through miRNAs microarray and bioinformatics analysis, we confirmed that miR-139 directly interacted with the 3′-untranslated region (3′-UTR) of CXCR4. Overexpression of miR-139 down-modulated CXCR4/p-Akt axis to attenuate invasion and migration of human breast cancer stem cells both in vitro and in vivo. Furthermore, miR-139 expression assessed by quantitative real-time PCR (qRT-PCR) in laser capture microdissected tumor samples significantly correlated with more advanced tumors in patients with breast cancer. Our findings provide support to account for the preferential role of miR-139 in interrupting breast cancer progression, identifying miR-139 as a potential biomarker in prediction of breast cancer invasiveness.

**Abstract:**

Elevated expression of C-X-C motif chemokine receptor 4 (CXCR4) correlates with chemotaxis, invasion, and cancer stem cell (CSC) properties within several solid-tumor malignancies. Recent studies reported that microRNA (miRNA) modulates the stemness of embryonic stem cells. We aimed to investigate the role of miRNA, via CXCR4-modulation, on CSC properties in breast cancer using cell lines and xenotransplantation mouse model and evaluated miR-193 levels in 191 patients with invasive ductal carcinoma. We validated miR-139 directly targets the 3′-untranslated region of CXCR4. Hoechst 33342 fluorescence-activated cell sorting (FACS) and sphere-forming assay were used to identify CSCs. MiR-139 suppressed breast CSCs with mesenchymal traits; led to decreased migration and invasion abilities through down-regulating CXCR4/p-Akt signaling. In lung cancer xenograft model of nude mice transplanted with human miR-139-carrying MDA-MB-231 cells, metastatic lung nodules were suppressed. Clinically, microdissected breast tumor tissues showed miR-139 reduction, compared to adjacent non-tumor tissues, that was significantly associated with worse clinicopathological features, including larger tumor size, advanced tumor stage and lymph node metastasis; moreover, reduced miR-139 level was predominately occurred in late-stage HER2-overexpression tumors. Collectively, our findings highlight miR-139-mediated suppression of CXCR4/p-Akt signaling and thereby affected mesenchymal stem-cell genesis, indicating its potential as a therapeutic target for invasive breast cancer.

## 1. Introduction

Breast cancer is the most common cancer type and the second leading cause of cancer mortality in women in Western countries, with approximately one in nine being affected over their lifetime [1]. Similar to the high prevalence in Western countries, its incidence rate in Taiwan has increased significantly over the last two decades; it is the most common cancer type detected and the fourth leading cause of cancer-related death in Taiwanese women since 2006 [the statistics of The Taiwan Cancer Registry (TCR), Ministry of Health and Welfare, http://tcr.cph.ntu.edu.tw/main.php?Page=N1]. Lymph node dissemination and colonization of distant organs by cancer cells, which acquire genetic and/or epigenetic alterations from primary tumor cells, has been examined in a specific subset of patients with higher metastatic potential; metastatic spread of the primary tumor accounts for over 90% of mortality in cancer patients [2,3]. Transformation of tumor cells to a malignant state comprises invasion and metastasis that are the key steps in cancer progression. Highly metastatic clones are reportedly sustained by subpopulation cells (SPs), also named cancer stem cells (CSCs), are capable of self-renewal and re-differentiation enabling the formation of new cancer colony settings that characterize the originality of tumors [4]. Cancers in most patients suffering from unfavorable outcomes are derived from organ-restricted CSCs that are identified by overexpression of embryonic stem cell-specific factors, including Nanog, Oct4, and CD133 [5]. CSCs phenotypically resemble normal progenitor cells and dysregulation of stemness-related signaling pathways, such as Wnt/β-catenin [6], Hedgehog [7], Notch [8], and PI3K/p-Akt [9], are often due to acquired genetic mutations and/or epigenetic changes in the tumor cells that in turn lead to metastasis and chemoresistance. Therefore, the association between genetic signature of CSC-biological traits and cancer prognosis can serve as a reliable marker for assessing clinical outcomes and developing therapeutic strategies for the disease.

Studies on tumor cell biology indicated that the interaction between C-X-C motif chemokine receptor 4 (CXCR4) and its chemokine ligand 12 (CXCL12; also known as stromal cell-derived factor 1, SDF-1) plays an essential role in cell oncogenesis and development of metastatic lesions in tumor [10]. Reportedly, upregulation of CXCR4 in conjunction with the specific extracellular matrix (ECM) components were conducted to enhance tumor cell viability, and migratory and invasive abilities in various cancers [11,12,13,14]. CXCR4 overexpression has been detected at the cDNA level by real-time quantitative reverse transcription PCR (RT-qPCR) analysis and at the protein level by immunohistochemistry and western blotting analyses in previous studies. These results indicated that CXCR4 participates in the malignant transformation of tumor cells in various human solid cancers [10,15], including breast carcinomas [16,17]. A recent study supported the key role of CXCR4 signaling in enhancing breast cancer tumorigenesis and invasiveness [18]. In addition, activating CXCR4/SDF-1 signaling promotes the maintenance of CSCs and acquisition of properties that ultimately contribute to drug resistance [19,20]. Oppositely, cultivation of breast cancer cells with CXCR4 antagonist, AMD3100, sensitized mesenchymal stem cells to cytotoxic drugs and reduced tumor metastatic burden [21]. Accordingly, CXCR4 is considered as a potential target not only for an earlier diagnosis of disease progression but also for therapeutic intervention.

MicroRNAs (miRNAs) are a family of small non-coding RNA molecules capable of down-modulating the translation of target genes by binding to their 3′-untranslated region (3′-UTR). They participate in a wide range of physiological and pathological processes, including cell cycle, development, differentiation, apoptosis, as well as angiogenesis, invasion, and metastasis of cancer cells [22,23]. Studies on breast CSC (BCSC) have revealed that miRNAs create multiple facets in maintaining self-renewal, initiation, and re-differentiation properties of CSCs, which correlate with poor clinical outcomes and drug resistance [24,25]. However, their role in modulating CXCR4 and subsequent downstream genes that affect the CXCR4/miRNA-transfected breast tumor cell phenotype has been rarely investigated. This has limited our understanding of miRNA function in BCSC processes through which CXCR4 increases the progression of breast cancer. In the current study, we explored the preferential role of miR-139 in interfering with breast cancer cell stemness by repressing the expression of CXCR4 protein. We also examined the mechanisms underlying miR-139-derived inhibition of CXCR4 signaling to decrease the invasion/migration of breast cancer both in vitro and in vivo. Given the molecular specificity of miR-139, we conducted a precise analysis using laser capture microdissection (LCM) and qRT-PCR (semi-quantifying) of LCM-collected tumor cells relative to surrounding non-tumor cells, which may aid in the prediction of breast cancer progression.

## 2. Results

### 2.1. Decreased Levels of miR-139 in Metastatic Breast Cancer Cell Lines 

**Figure 1:**
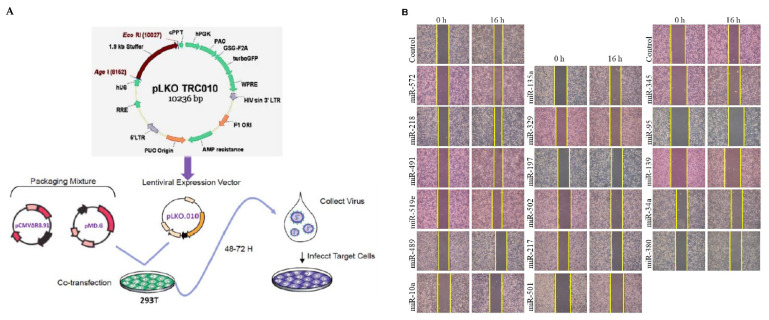

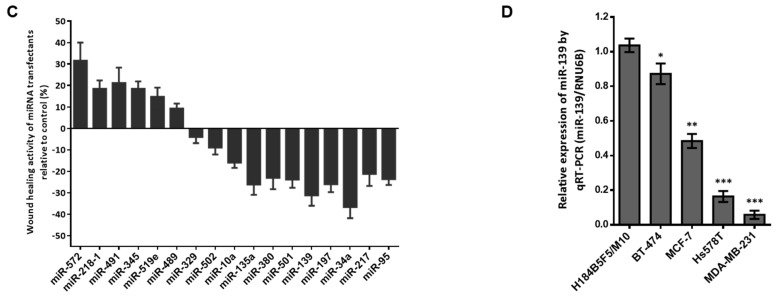
Selection of candidate miRNAs by wound healing assays. (**A**) Cloning strategy of plasmid pLKO10 carrying different primary-miRNA templates in MDA-MB-231 cells. (**B**) Representative images for wound healing assay in tumor cells at 0 and 16 h after scarification. (**C**) Relative quantification of wound healed area in MDA-MB-231 cells carrying miRNA of interest compared with the pLKO10 control group. Data are expressed as the mean ± SD of three experiments. (**D**) miR-139 was significantly decreased in metastatic breast cancer cell lines (Hs578T and MDA-MB-231) or non-metastatic breast cancer (BT-474 and MCF-7) cell lines as compared to normal breast epithelial (H184B5F5/M10). Expression levels of miR-139 were normalized to *RNU6B*. * *p* < 0.05, ** *p* < 0.01, and *** *p* < 0.001.

We previously identified 52 miRNAs that were significantly dysregulated, with a greater than two-fold expression change, in cells isolated from the primary tumor site in patients with and without lymph node metastasis (LNM) [26]. Hereby, by using the lentivirus-based expression vector pLKO10 (National RNAi Core Facility, Academia. Sinica, Taipei, Taiwan) and Trans-Lentiviral^TM^ Packaging System (Figure 1A), we examined the inhibitory effect of 17 of these candidate miRNAs (primer set were shown in Table S1), displaying reduced expression, on breast cancer migration using wound healing assay (Figure 1B). The reduced migration activity was quantified as >30% for two miRNAs, miR-34a and miR-139-5p (miR-139) (Figure 1C). The tumor suppressive functions of miR-34a have been well-established in several cancer types [27,28,29], including breast cancer [30]; however, as the anti-metastatic function of miR-139 in human breast cancer remains scantly reported, we explored the effect of miR-139 on breast cancer progression in vitro. As shown in Figure 1D, the qRT-PCR results of human breast cancer cell lines revealed that miR-139 expression levels were substantially lower in highly invasive Hs578T and MDA-MB-231 breast cancer cells as compared to that in non-invasive MCF-7 and BT-474 breast cancer cells as well as non-malignant mammary epithelial cell line H184B5F5/M10.

### 2.2. CXCR4 Gene as a Direct Target of miR-139

**Figure 2:**
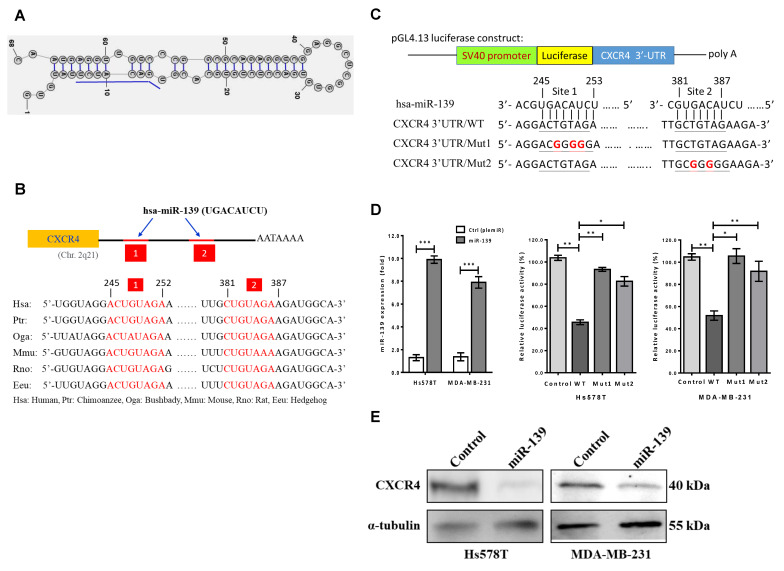
CXCR4 as a direct target of hsa-miR-139-5p. (**A**) The stem-loop sequences hsa-miR-139-5p (miR-139) using RNAstructure, Version 6.2 and sequences in the mature miR-139 (underlined in blue) are shown. (**B**) Prediction of the targeting sequence for miR-139 within the 3′-UTR of CXCR mRNA. Two binding motifs in the 3′-UTR of CXCR4 gene for miR-139 (red) are shown across different vertebrate species. (**C**) Schematic representation of the luciferase reporter constructs showed a mismatch of the miR-139 binding sequence that are underlined at site 1 (CXCR4 3′-UTR/Mut1) and site 2 (CXCR4 3′-UTR/Mut2), respectively. (**D**) The dual-luciferase reporter (DLR™) assay was evaluated in breast cancer cell lines expressing the constructs designed in (**C**). Firefly luciferase activity was normalized to Renilla luciferase activity and compared with the cells overexpressing miR-139 or empty vector (control). Data are presented as the mean ± SD of three independent experiments. * *p* < 0.05, ** *p* < 0.01. (**E**) A significantly decreased expression of CXCR4 was observed in both invasive breast cancer cell lines transfected with miR-139. α-tubulin served as the loading control.

Stepwise, we attempted to identify the downstream target gene of miR-139 that promote breast cancer cell invasiveness. Initially, the single-stranded stem-loop RNA precursors containing hairpin structures and the seeding sequences of premature miR-139 were analyzed using RNAstructure 6.2 software (Figure 2A). Based on three online miRNA target prediction programs, miRBase, miRWalk2.0, and TargetScan7.0, we postulated CXCR4 as the direct target of miR-139. CXCR4 contains two conserved RNA sequence domains in its 3′-UTR that have complementarity with miR-139 (Figure 2B). The pGL4.13/dual-luciferase reporter assay revealed that miR-139 significantly reduced the luciferase activity of the pGL4.13 carrying full-length CXCR4 3′-UTR (CXCR4 3′-UTR-luc, WT) by more than 55% compared to the respective control group (*p* < 0.01) (Figure 2C). This reduced luciferase activity was restored in presence of pGL4.13 reporter construct containing mutations in the 3′-UTR of CXCR4, either in site 1 (Mut1) or site 2 (Mut2) (Figure 2D). In addition, repression of CXCR4 protein was more prominent (>70%) in miR-139-transfected Hs578T and MDA-MB-231 cells than control cells (Figure 2E).

### 2.3. miR-139 Inhibited Breast Cancer Invasiveness by Suppressing CXCR4/p-Akt Signaling

**Figure 3:**
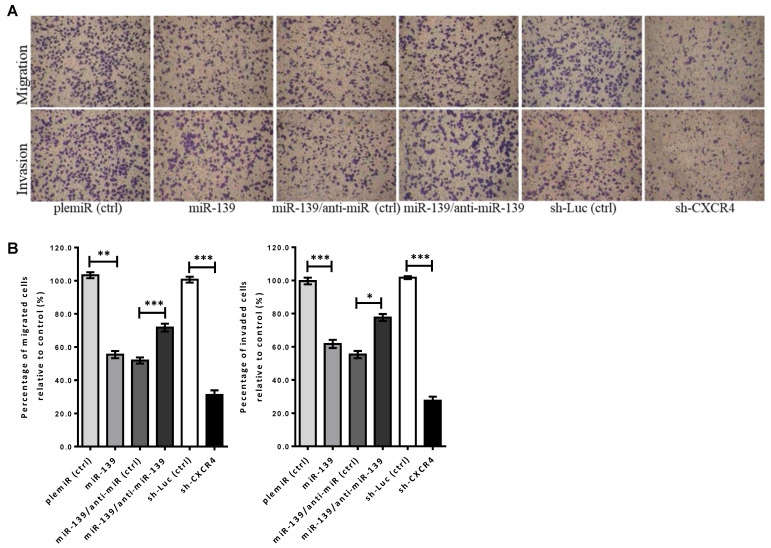

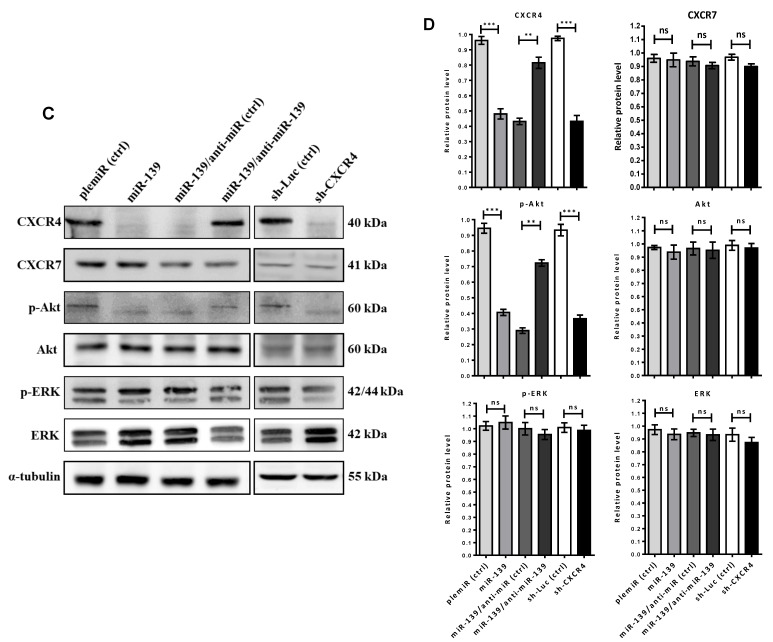
miR-139 inhibits breast cancer cell aggressiveness by targeting CXCR4. (**A**) Representative micrographs showed migration (upper panel) and invasion (lower panel) filter membranes after crystal violet staining in Boyden chamber assay for lentiviral miR-139-transfected MDA-MB-231 breast cancer cells that were co-treated with miR-139 inhibitor (anti-miR-139) or negative control [anti-miR (ctrl)]. Besides, knockdown of CXCR4 gene (shCXCR4) was compared with the control group [sh-Luc (ctrl)]. (**B**) Quantitative analysis of migration and invasion are expressed as mean ± SD of three independent experiments. (**C**) Reduced levels of CXCR4 and phosphorylated-Akt in miR-139-transduced or shCXCR4-MDA-MB-231 breast cancer cells were shown by western blot analysis. (**D**) Quantitative analysis of the western blot results; α-tubulin was used as internal control for protein loading. ns, not significant; * *p* < 0.05, ** *p* < 0.01, and *** *p* < 0.001.

The CXCR4/CXCL12 axis is known to play an integral role in promoting cancer cell progression via phosphorylation of Akt at Ser473 (pAkt^Ser473^) that induces epithelial-to-mesenchymal transition (EMT) [31]. Thus, we examined the mechanism underlying miR-139-mediated inhibition of breast tumor cell metastatic phenotype. Results from Boyden chamber assays (Figure 3A) revealed decreased invasion and migration abilities in the miR-139-transfected MDA-MB-231 cells by >40% compared to that of mock-transfected controls (Figure 3B). Besides, the impaired invasive potential due to CXCR4 repression by the treatment of miR-139 in breast cancer cells was confirmed via using short hairpin RNAs (shRNAs) against CXCR4 (sh-CXCR4) (Figure 3A,B). Mechanistically, miR-139 attenuates CXCR4 signaling to inhibit breast cancer invasiveness via inactivation of phosphorylated-Akt (pAkt^Ser473^), rather than phosphorylated-ERK signaling, in the miR-139-transduced MDA-MB-231 breast cancer cells (Figure 3C,D).

### 2.4. miR-139 Decreased the Stemness of Breast Cancer Cells

**Figure 4:**
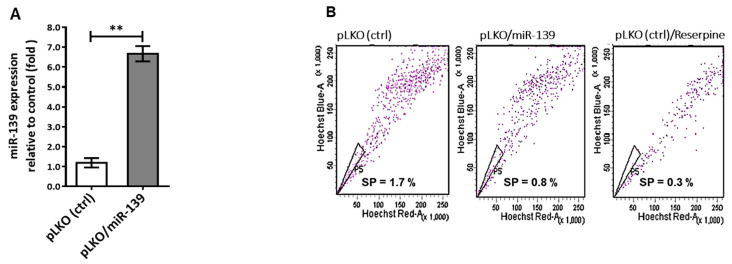

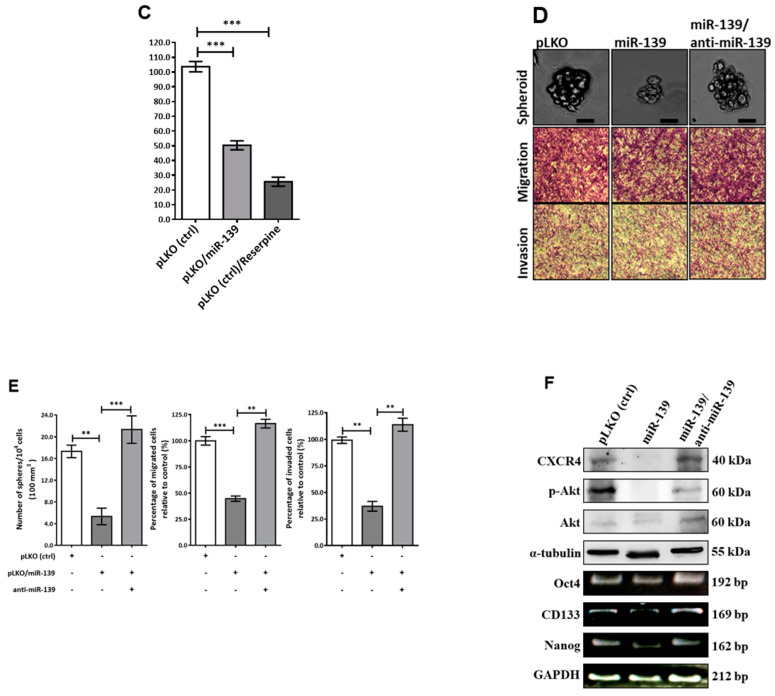
MiR-139 targeting CXCR4 suppressed stemness of breast cancer cell. (**A**) Lentivirus plasmid vector (pLKO) alone or with miR-139 (pLKO/miR-139) were transfected into MDA-MB-231 cells and TaqMan-based real-time PCR was used to quantify relative expression of miR-139 along with *RNU6B*. (**B**) Side-population cells (SPs), known as CSCs, were identified using ABCG2 inhibitor (reserpine) and sorted by FACS technique in combination with Hoechst 33342 dye. (**C**) Percentage of SPs generated from MDA-MB-231 cells that were transfected with miR-139 or negative control, and their miRNA expression levels were assessed with qRT-PCR in (**A**). (**D**) Tumorsphere formation of SPs (upper panel). Scale bar, 50 µm; representative images of migration (middle panel) and invasion (lower panel) for BCSCs with different treatments to determine the inhibitory effect of miR-139 on BCSC stemness. Images were taken at 100× magnification. (**E**) Spheres were counted in 1 × 10^3^ cells each: MDA-MB-231 cells containing pLKO-miR-139 and pLKO-miR139 cell treated with the miR-139 inhibitor (miR-139/anti-miR-139) as indicated. The invading tumor cells were counted in five random fields of view. (**F**) Western blot analysis (expression of CXCR4 and p-Akt proteins) and qPCR results of mRNA transcripts encoding CD133, Nanog, and Oct4 in miR-139-transfected MDA-MB-231 BCSCs compared to that in miR-139/anti-miR-139 BCSCs. ** *p* < 0.01, *** *p* < 0.001.

The multi-step transformation of tumor cell malignancy involves pluripotency reprogramming, and CXCR4 is reportedly involved in cell pluripotency that triggers cell renewal, chemotaxis, and re-differentiation, in a cancer niche [32]. Thus, our proposed mechanism for miR-139/CXCR4 conducted inhibition of tumor cell aggressiveness could be endorsed by repression CXCR4-mediated cancer cell stemness. Based on our previous study experience [33], we established a lentiviral vector pLKO stably expressing miR-139 transcript using Trans-Lentiviral^TM^ Packaging System. As shown in Figure 4A, miR-139 transcript expression increased 7-fold in pLKO/miR-139-transfected MDA-MB-231 cells compared to that in control cells. CSCs were quantitated using Hoechst 33342 dye and fluorescence-activated cell sorting (FACS) of cancer cells treated with 20 µM reserpine (Figure 4B). Based on this sorting process, the number of BCSCs was significantly lower than 50% in pLKO-miR-139/MDA-MB-231 cells compared to the control group (Figure 4C). Further, to determine the involvement of miR-139 in BCSC proliferation, we assessed the cell cycle using flow cytometry. The results indicated that addition of miR-139 had no significant effect on the distribution of BCSCs during different phases of cell cycle when compared with the mock group (Figure S1). Contrarily, transfecting MDA-MB-231 cells with miR-139 reduced the number of tumorsphere-forming cells by >70% (Figure 4D,E), which in turn significantly reduced the transcription levels of stemness markers, *Oct4*, *Nanog*, and *CD133* genes (Figure 4F). Also, Boyden chamber assay revealed >70% decrease in cell migration and invasion in miR-139-transfected MDA-MB-231 cells compared to control cells (Figure 4D,E), along with decreased expression of CXCR4 and p-Akt proteins using western blot analysis (Figure 4F). Conversely, miR-139-transfected MDA-MB-231 cells co-treated with an inhibitor against miR-139 (anti-miR-139) regained cell migration and invasion properties (Figure 4E). Collectively, these data indicated that miR-139 down-modulates CXCR4 to show decreased re-differentiation property of stemness, leading to inhibition of tumor metastasis and aggressiveness.

### 2.5. miR-139 Reduced BCSC Through Down-Modulation of CXCR4/p-Akt Signaling

**Figure 5:**
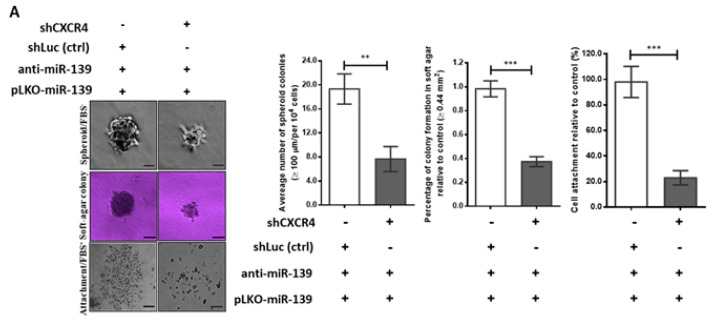

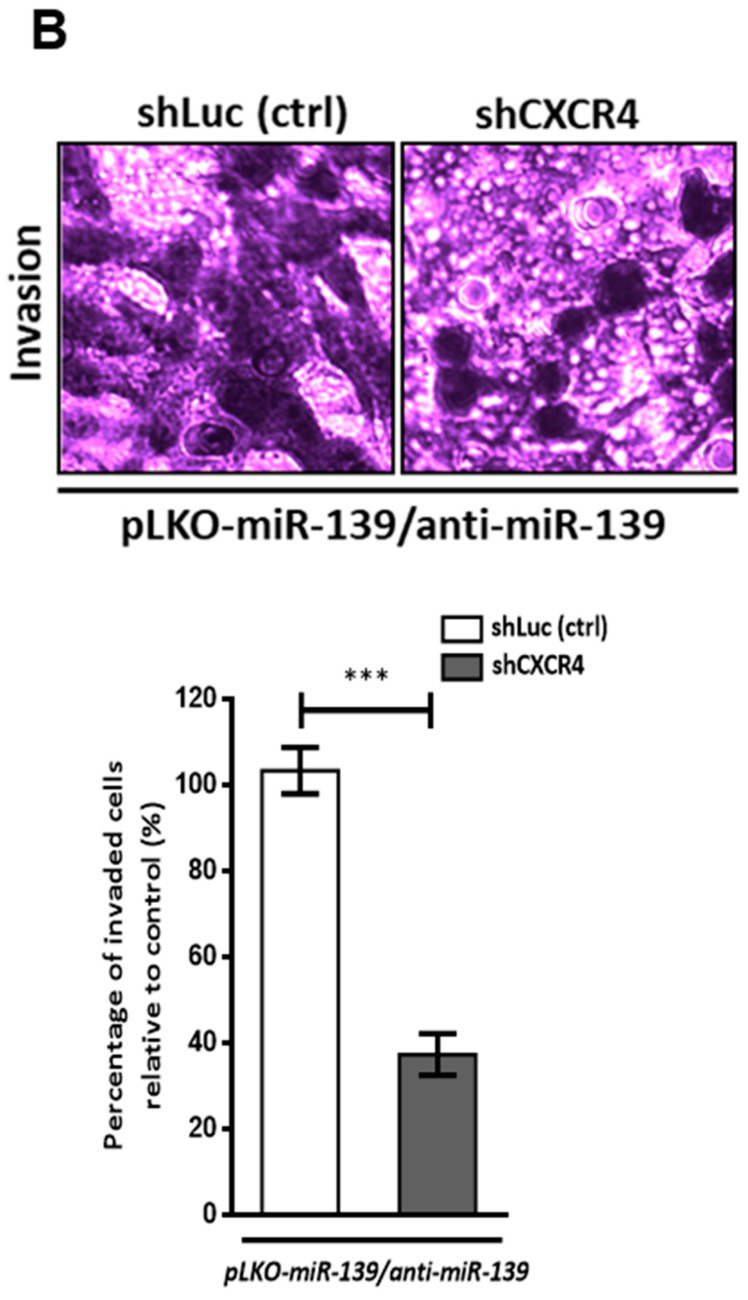

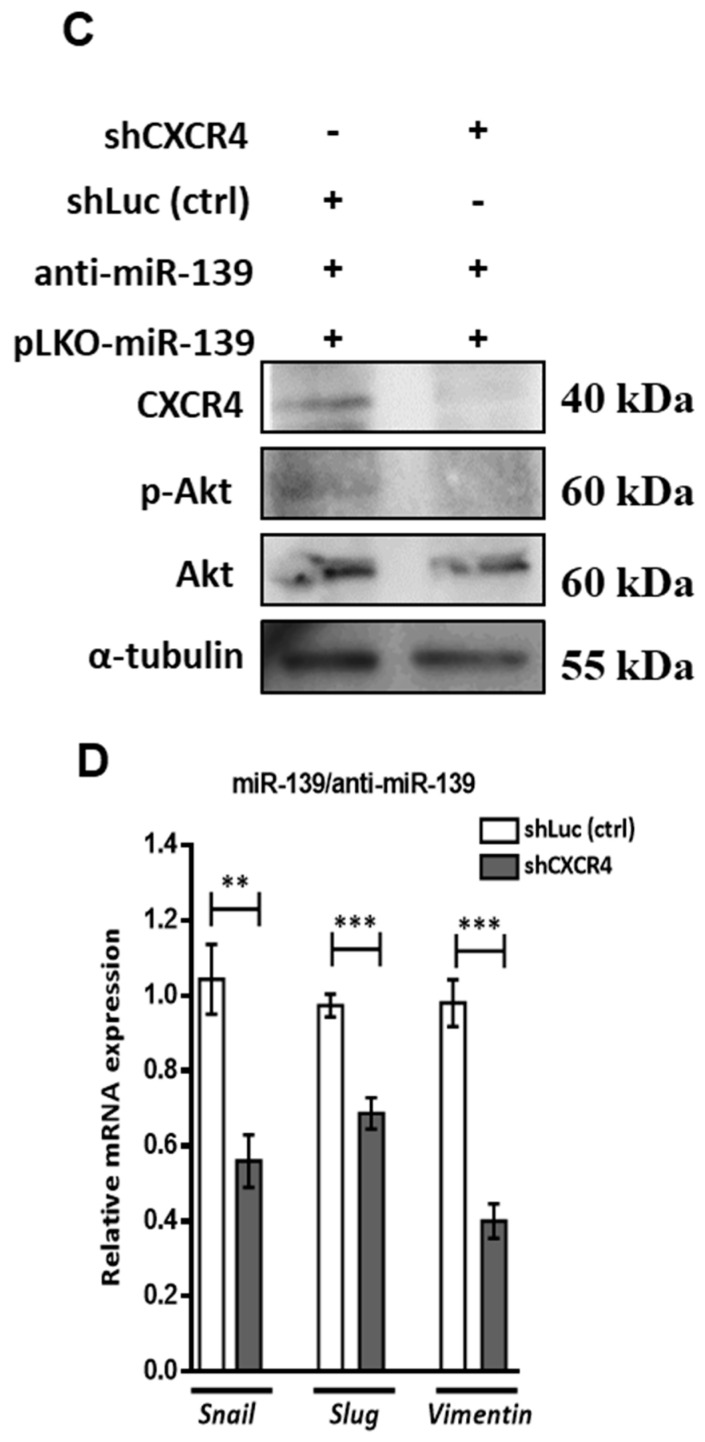

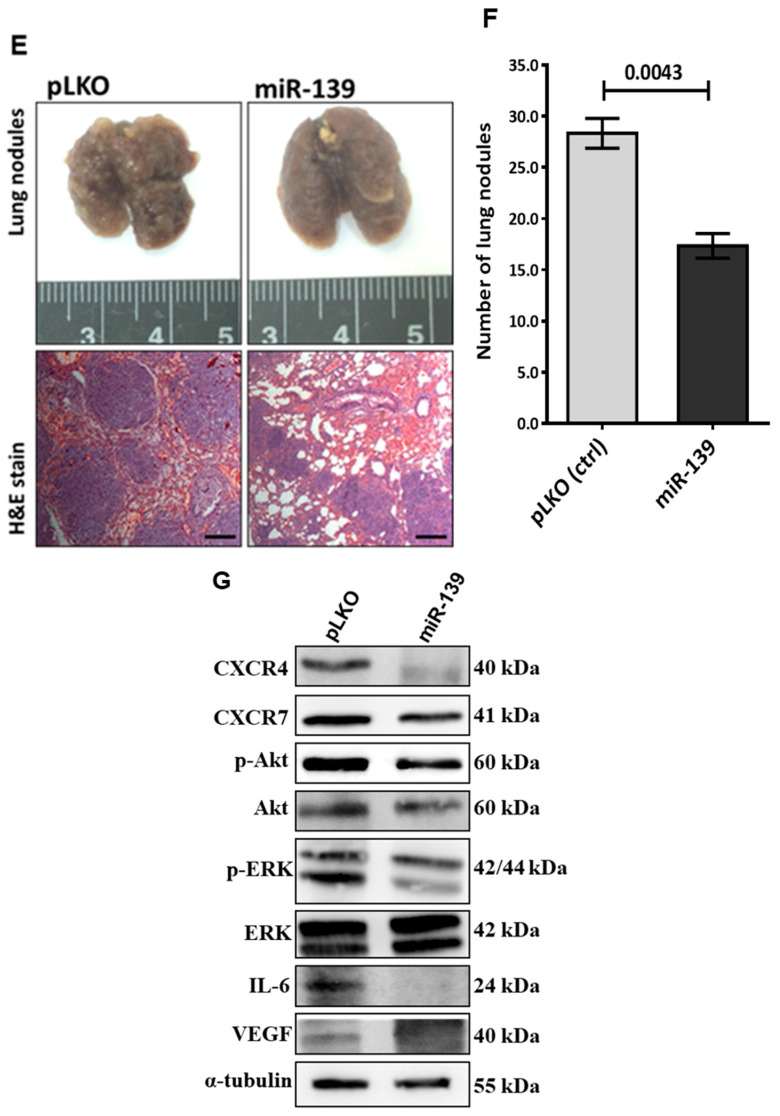
MiR-139 inhibits breast cancer cell stemness and metastatic lung colonization of breast cancer xenografts via down-modulation of CXCR4. (**A**) Representative images and histograms show the stemness characteristics, including the spheroid cell aggregation, colony formation in soft agar, and cell attachment behavior in MDA-MB-231 cells treated as indicated. Scale bar, 50 µm. (**B**) Representative photos of Boyden chamber assay. Invasion capacity was assessed in BCSCs carrying pLKO-miR-139 co-transfected with miR-139 inhibitor and the negative control [shLuc (ctrl)] or shCXCR4, as indicated. cancer cells transfected with different shRNAs as indicated. (**C**) Western blot results of CXCR4, p-Akt, and Akt in BCSCs carrying pLKO-miR-139 co-transfected with miR-139 inhibitor in combination with different shRNAs as indicated. (**D**) Histograms show mesenchymal markers (Snail, Slug, and vimentin) in MDA-MB-231/pLKO-miR-139 cells co-treated with miR-139 inhibitor (anti-miR-139) and different shRNAs as indicated. Data are presented as the mean ± S.D. of three independent experiments. ** *p* < 0.01, *** *p* < 0.001. (**E**) Representative lungs and HE staining of metastatic tumor lung tissues from mice five weeks after tail vein injection of MDA-MB-231 cells carrying miR-139 or pLKO vector (control) are shown. Scale bar, 100 µm. (**F**) Number of metastatic nodules in lungs of mice (*n* = 5 per group). (**G**) Five weeks post-xenotransplantation, lung tumors were excised and sectioned. Western blot analysis of the proteins of interest examined in lung tumors carried pLKO-miR139/MDA-MB-231 cells or control group as shown. α-tubulin was used as an internal control.

As CXCR4-induced Akt phosphorylation upregulates VEGF expression in tumor metastasis by enhancing mesenchymal proteins [34], we explored the regulatory action of miR-139 on CXCR4/p-Akt axis with respect to stemness and invasiveness properties of breast cancer cell linking VEGF-mediated EMT. Thus, treatment with miR-139 inhibitor (anti-miR-139) in pLKO-miR-139-transduced MD-MBA-231 cancer cells restored their stemness characteristics, such as spheroid cell aggregation, colony formation in soft agar, and cell attachment behavior; these features indicating increased stemness were abrogated on silencing the *CXCR4* gene (*shCXCR4*) (Figure 5A). Besides, >60% reduction in cancer cell invasion was observed in CXCR4-silenced pLKO-miR-139-transduced MDA-231 cells as compared to the control shRNA-treated cells, suggesting that the restored tumor invasiveness because of anti-miR139 treatment in these cells was significantly decreased by knockdown of CXCR4 and phosphorylated Akt proteins (Figure 5B,C). Snail and Slug (zinc finger proteins) are direct transcriptional factors that enhance the EMT pathway in highly invasive and metastatic cancer [35]; thus, we examined the expression of these two upstream transcription factors and a downstream mesenchymal marker (vimentin) in relation to miR-139 in BCSCs. As expected, treatment with an miR-139 inhibitor (anti-miR-139) in miR-139-transfected MDA-MB-231 cells counteracted the suppressive function of miR-139 in repressing CXCR4 protein expression, which enhanced cell migration and invasion properties via CXCR4-induced increased genesis of BCSCs (Figure 4D,E). In contrast, the above enhanced migration and invasion abilities in miR-139-transfected MDA-MB-231 cells treated with anti-miR-139 were significantly prohibited by treatment with shCXCR4 (Figure 5B), coinciding with reductions in the Snail, Slug, and vimentin transcripts during EMT (Figure 5D). These findings confirm that miR-139 inhibits the stemness and invasiveness of human breast cancer cells by repressing CXCR4 signaling to decrease EMT.

To understand the impact of miR-139 on metastatic suppression in vivo, we established lung metastases in a xenograft model by transplanting breast cancer cells (5 × 10^6^ cells/mouse) with or without miR-139 into the tail vein of female BALB/c-nude mice. After 5 weeks of incubation, mice were sacrificed, and their lungs were dissected to evaluate tissue morphology by HE staining (Figure 5E). As shown in Figure 5F, we found that the average number of pulmonary metastatic nodules in mice injected with MDA-MB-231/pLKO-miR-139 cells was significantly lower than that in the control group (*p* < 0.01). Western blotting analyses demonstrated markedly decreased expression levels of CXCR4 and p-Akt proteins in pLKO-miR-139-transfected MDA-MB-231 cells (Figure 5G). As reported in previous, CXCR4/p-Akt signaling correlated unfavorable outcome of acute myeloid leukemia patients via upregulation of vascular endothelial growth factor (VEGF) and IL-6 gene expression [36]; in this study, we found decreased protein levels of VEGF and IL-6 coincided with CXCR4 reduction in the xenograft-transplantation model of breast cancer cells carrying miR-139 (Figure 5G).

### 2.6. Prognostic Significance of miR-139 in Patients with Breast Cancer

**Figure 6:**
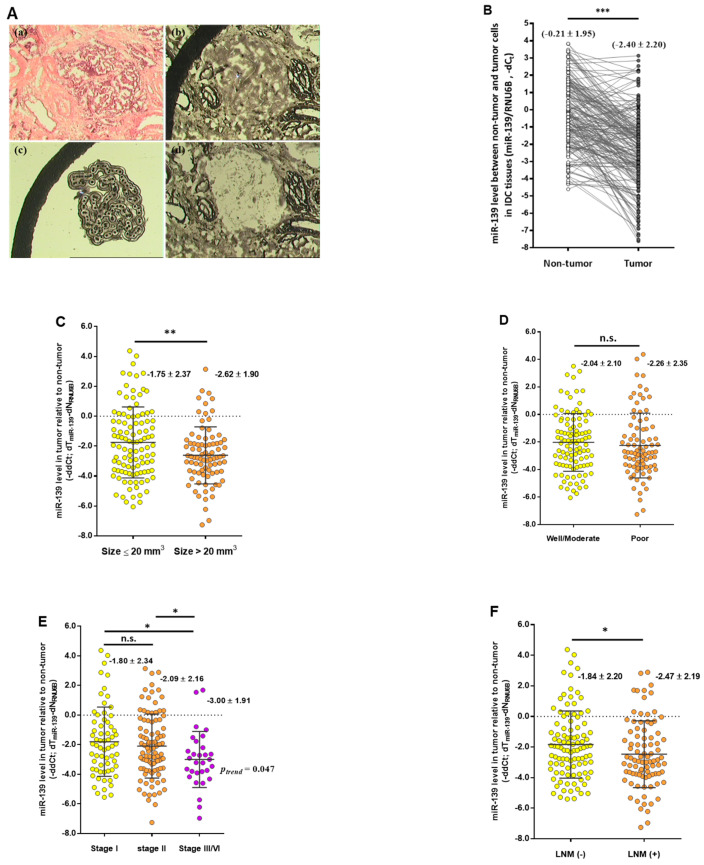
Decreased expression of miR-139 is associated with poor clinicopathological features. (**A**) Representative images of microdissected cancer cells with laser capture microdissection (LCM) technique. (**a**) Hematoxylin and eosin staining and (**b**) histologic staining with Ambion’s RNAqueous-Micro kit (Ambion Inc., Austin, TX, USA) of the breast cancer specimen; LCM-treated breast cancer cells (**c**) and after (**d**) microdissection are shown, respectively. (**B**) The relative expression of miR-139, normalized to *RNU6B*, was detected by qRT-PCR from paired LCM-collected tumor tissue and adjacent normal breast epithelium of 191 patients with IDC. (**C**)–(**F**) Expression level of miR-139 from LCM-captured cancerous and adjacent noncancerous cells was measured by qRT-PCR (TaqMan Advanced miRNA Assay probe). Each dot represents a case, the middle line marks the mean, and the upper and lower depict the borders of the 25 and 75% quartiles. Decreased expression levels of miR-139 in primary tumor tissues correlate with aggressive tumor phenotypes, such as larger tumor size (**C**), histologic grades (**D**), advanced stages (**E**) or LNM (**F**) using the Mann-Whitney U test. * *p* < 0.05, ** *p* < 0.01, *** *p* < 0.001.

Since miR-139 was found to downregulate CXCR4 and thus inhibit tumor cell aggressiveness both in vitro and in vivo, we next measured miR-139 levels in women diagnosed with invasive ductal carcinoma (IDC). To ensure the assayed tissue samples containing a homogenous population of cells, including more than 95% tumor cells and paired adjacent noncancerous cells to precisely measure the miR-139 expression level, laser capture microdissection (LCM) technique was done on routinely immunostained slides using a PixCell laser capture microscope (Arcturus Engineering, Inc., Santa Clara, CA, USA) (Figure 6A). Expression analysis using comparative CT method revealed that the mean expression level of miR-139 in microdissected tumor cells was significantly lower than that in corresponding non-tumor cells (−2.40 vs. −0.21; *p* < 0.0001; Figure 6B). In addition, comparative quantification of miR-139 levels performed for the individual patients, the normalized mean expression values of miR-139 (relative to U6B small nuclear RNA gene, *RNU6B*) detected in primary tumor tissues were significantly lower than those in the adjacent non-tumor tissues, correlating with more advanced tumors, such as large tumor size (>2 mm^3^), late-stage or lymph node-positive breast tumor (Figure 6C–F). Further, we determined the cutoff for defining reduced expression of miR-139 level as four-fold decrease (−∆∆Ct = 2.2; 2^−2.2^ = 0.22), which was used to classify patients with low miR-139 expression in association with clinicopathological features in the following analysis. As shown in Table 1, patients with decreased expression of miR-139 correlated with large tumor size (*p* = 0.044), advanced clinical stage (stage III/IV vs I/II, *p* = 0.013), and LNM (LNM-positive vs. LNM-negative, *p* = 0.023), respectively. Because hormone receptor status of estrogen receptor (ER), progesterone receptor (PR), and human epidermal growth factor receptor 2 (HER2) are routinely evaluated for predicting prognosis and determining treatment for breast carcinoma [37], upon stratification of patients according to status of ER, PR or HER2, we performed an association analysis to assess low miR-139 expression level and clinicopathological feature of tumors. Under the same categorization in the multivariate logistic regression analysis, miR-139 expression was inversely associated with advanced clinical stages and LNM in ER-negative or PR-negative tumors (*p* < 0.05). On the contrary, association between decreased level of miR-139 and late-stage tumor was observed in a subgroup of patients with HER2-positive breast cancer (*p* = 0.026) (Table 2).

## 3. Discussion

There has been growing interest in exploring the innate ability of miRNAs to govern the aggressiveness of cancer cells by modulating a vast range of physiological responses during tumor progression. Thus, understanding the molecular signatures of miRNA-targeted tumor suppressor genes or oncogenes would not only serve as a prognostic indicator but also help develop more effective therapeutics for the disease. In this study, we validated the potential function of miR-139/CXCR4 in p-Akt inactivation that influence the advanced disease phenotype in breast cancer cells. Further, the xenotransplantation of miR-139-carrying human breast cancer cells in mice significantly decreased the number of metastatic lung nodules. Moreover, consistent with previous reports in different types of cancers [38,39], miR-139 level was lower in breast tumor cells as compared to non-tumor cells in our patient cohort. Reduced miR-139 levels correlated with larger tumor size, late-stage cancer, and the presence of LNM in IDC. These findings provide evidence to support miR-139 as a reliable biomarker that facilitates a priori prediction of advanced tumor phenotypes in women with breast cancer.

MiRNAs principally bind to mRNAs via less-than-perfect complementarity with their 3′-UTR; thus, a single miRNA can target several mRNAs to modulate a robust physiological response. For example, miR-139 can suppress endometrial cancer cell growth and migration via down-regulation of HOXA10 [40], restore docetaxel-chemosensitivity by targeting Notch1 [41], decrease tumor cell over-proliferation and EMT process in metastatic prostate and colorectal cancer cells [42,43]. miR-139 inhibits proliferation through translational repression of COL11A1, leading to the upregulation of Bax and activation of Caspase 3-mediated apoptosis of breast cancer cells [44]. Based on these observations, miR-139 was demonstrated to exert its tumor-suppressive roles by inhibiting cancer cell over-proliferation and metastasis and promoting apoptosis of cancer cells and may serve as a prognostic marker in predicting the aggressiveness of breast cancer. Interestingly, E-cadherin and N-cadherin expression switch are key proteins that promote tumor cell migration and invasion during EMT. E-cadherin loss in epithelial cells and N-cadherin overexpression in mesenchymal cells are regulated by the Notch1-Slug signaling axis [45]. As Notch1 is a direct target of miR-139 [41], further studies are needed to determine the additional role of miR-139-mediated down-modulation of the Snail/Slug-EMT axis in increasing E-cadherin levels to inhibit the invasive mesenchymal phenotype of breast cancer by targeting Notch1. Hereby, we conducted overexpression of miR-139 to reduce BCSC genesis by down-regulating CXCR4/p-Akt axis, leading to decreased invasion and migration abilities of breast cancer cells. The multistep development consisting tumor initiation, cancer progression and drug-resistance has been reportedly inhibited by miRNAs that suppress self-renewal and re-differentiation of CSCs via down-modulating oncogenic signaling pathways in human cancer [36,46,47,48]. Along similar lines, our results showed that miR-139-mediated CXCR4 reduction correlated with decreased levels of VEGF and IL-6 in the xenograft-transplantation model of human breast cancer. To our knowledge, the present study is the first of its kind investigation into the link between impediment of miR-139, stemness of CSCs, and resulting phenotypic changes with respect to the metastatic potential and prognostic significance in human breast cancer.

Based on the status of hormone receptors (ER and PR) and HER2, four principal molecular subtypes [luminal-A, -B, HER2-enriched and triple-negative breast cancer (TNBC)] have been characterized and are routinely used to predict prognosis and optimize adjuvant therapy in breast cancer [37]. In this study, we identified miR-139 as a potential biomarker that may play a predictive role in breast cancer with a large tumor size, advanced clinical stage, and presence of nodal metastasis of breast tumors. However, as a prognostic surrogate for discriminating breast cancer subtypes, the mean expression level of miR-139 is lower in HER2-enriched breast tumors but shows no significant difference in frequency compared to other breast cancer subtypes (Supplementary Figure S2 and Table S2). In addition, the effect of decreased miR-139 levels on the clinical outcomes of breast cancer were evaluated by comparing the overall 5-year survival of each patient; ~95% (94.8%; 181 of 191) of our patients with cancer were analyzed. We found that decreased miR-139 level was not significantly associated with poor OS according to Kaplan-Meier analysis (log-rank test) with Bonferroni correction and Cox proportional hazards regression analysis (Supplementary Figure S3A). Interestingly, based on the Kaplan-Meier Plotter website (http://kmplot.com/analysis/), the association between miR-139-5p expression and the OS of patients with breast cancer has been inconsistent in observational studies such as in the database of METABRIC and independent cohorts (Supplementary Figure S3B,D). These positive and inverse associations between miR-139 and patient survival may be related to the inclusion of various ethnic groups in different studies. A limitation of gene biomarkers and clinical outcomes in cancer cohort studies is the different follow-up times and molecular subtyping of patients with cancer. Thus, multiple and larger cancer cohorts should be evaluated by meta-analysis to reinforce the role of the differential expression of genes, such as the miR-139 expression level, to estimate the correlation between survival and breast cancer subtype.

In this study, we found that miR-139 overexpression influences breast cancer cell migratory and invasive capabilities in both studies in vitro and in vivo. Decreased level of miR-139 was significantly associated with aggressive phenotype of breast cancer. We also observed a significant correlation between decreased miR-139 level and the poorer clinicopathological feature of breast cancer upon patient stratified by status of ER, PR, or HER2. A consistent trend of the associations of lower miR-139 expression with more advanced tumor phenotypes was observed in clinical samples upon subgroup analyses based on molecular subtypes, however, these associations are not statistically significant due to limited sample size (Supplementary Table S2). Taken together, the results from clinical data could partially supported our hypothesis that miR-139 reduction as a predictive biomarker of aggressive breast cancer. More importantly, our awareness remained toward rewiring the possible gene effectors involving in elevated level of CXCR4 during malignant progression of breast cancer. Reportedly, CXCR4 expression was concurrently increased with VEGF and IL-6 levels in stem cell chemoresistance of acute myeloid leukemia [36]. HER2-mediated CXCR4 expression can enhance breast tumor cell invasion and lung metastasis [49]. The molecular mechanism underlying the protective effect of ER via transcriptional repression of HER2 has been proved in HER2-overexpressing breast cancer cells [50]. In addition, Bao et al. [51] indicated an inverse correlation between HER2 and miR-139 expression in gastric cancer; the interaction between HER2 and CD44-enhanced histone deacetylation in the miR-139 promoter region upregulates CXCR4 expression, leading to gastric tumor progression and subsequently metastasis. Therefore, those possible genes effectors involving in elevated level of CXCR4 may hinder the pleiotropic roles of miR-139 in associating with poor patient survival. A further clinical investigation with a larger sample size that provides more information regarding *CXCR4* expressions and gene transcripts of those enhancers, such as *VEGF*, *IL-6* and *CD44*, is worthy to do. Notably, the inherent heterogeneity of breast tumor cells and evolutionary variations may lead to inconsistencies in molecular typing between primary and metastatic lesions. It has been interpreted that undergoing of developmental processes of CSCs in terms of promotion of cell tumorigenicity, activation of tumor metastasis and evasion of death execution events are regulated by distinct miRNA patterns [52]. Therefore, in the era of precision medicine, a comprehensive understanding of gene/miRNA profiles that are distinguished by the molecular subtype of breast cancer is of particular importance in identifying the determinant miRNA as a therapeutic target for developing more effective strategy for breast cancer management.

## 4. Materials and Methods

### 4.1. Cell Lines and Cell Culture

Human breast cancer cell lines Hs578T, MDA-MB-231, MCF-7, and BT-474 and the human mammary epithelial cell line H184B5F5/M10 were purchased from the Bioresource Collection and Research Center (BCRC, Hsinchu, Taiwan). These cell lines were cultured in Dulbecco’s Modified Eagle Medium (Sigma, St. Louis, MO, USA) with 0.1 mM sodium pyruvate, 10% fetal bovine serum (Gibco, BRL, Grand Island, NY, USA), 2 mM l-glutamine, 100 U mL^−1^ penicillin, and 100 µg mL^−1^ streptomycin (Sigma-Aldrich, Saint Louis, MO, USA). The cells were maintained at 37 °C in a humidified incubator with 5% CO_2_.

### 4.2. Cancer Stem Cell Sorting

CSCs were sorted as previously described [53]. Briefly, dissociated breast cancer cells were suspended at a concentration of 2 × 10^6^ cells mL^−1^ in SP medium (calcium- and magnesium-free Hanks’ balanced salt solution containing 2% fetal bovine serum, 1% penicillin/streptomycin and 10 mM HEPES). Hoechst 33342 (5 μg mL^−1^) (Sigma-Aldrich) was added to SP medium and intermittently mixed for 90 min at 37 °C. Reserpine (50 μM final concentration; Sigma-Aldrich), was introduced prior to adding Hoechst 33342 to inhibit dye efflux. The cells were maintained on ice in the dark for FACS, after which SP and non-SP fractions were sorted (FACS Digital Vantage/Diva cell sorter; BD Biosciences, San Jose, CA, USA). Hoechst 33342 dye was excited at 355 nm and the dual-wavelength fluorescence was measured by emission for blue at 450 nm and red at 675 nm.

### 4.3. Cultivation and Characterization of CSCs

For serial passage of spheroid cells, single cells were cultured in low-attachment 6-well plates (Corning, Inc., Corning, NY, USA), and the cell density at each passage was 5 × 10^3^ cells mL^−1^ in serum-free medium. After cultivation of the SP cells, total RNA was extracted from the cells using an RNA extraction kit (RNeasy) (QIAGEN, Hilden, Germany). The RNA (1 μg in a volume of 5 μL) was reverse-transcribed for 70 min at 42 ℃ using 5 units of Superscript II reverse transcriptase (Gibco-BRL) and 10 mM random primers of oligo(dT) primer (Promega, Madison, WI, USA) in a reaction volume of 20 μL. We used glyceraldehyde 3-phosphate dehydrogenase (*GAPDH*) as an internal standard for RT-PCR screening of CSC markers, including *Oct4*, *Nanog*, and *CD133*. Human *Oct4*, *Nanog*, and *CD133* cDNA fragments were amplified using the following primer sets. Oct4_F: 5′-CGCTTTGAGGGTCTGCAGCTT-3′ and Oct4_R: 5′-GAACAAATTCTCCAGGTTGCC-3′ (192 bp); Nanog_F: 5′-TAGCAATGGTGTGACGCAGA-3′ and Nanog_R: 5′-GCTCCAGGACTGGATGTTCTG-3′ (169 bp), and CD133_F: 5′-ACAATTCACCAGCAACGA-3′ and CD133_R: 5′-GGAAGTATTGTTTGTGATG-3′ (162 bp). PCR products were separated by 1.8% agarose gel electrophoresis, stained with ethidium bromide, and visualized under an ultraviolet light.

### 4.4. Construction of miR-139-Transfected Breast Tumor Cells

The lentivirus vector pLemiR (control) or pLemiR-139 (plemiR carried hsa-miR-139) was packaged with the Trans-Lentiviral^TM^ GIPZ Packaging System (Open Biosystems, Huntsville, AL, USA). A puromycin-resistant marker was used to select against non-transduced cells to amplify miR-139 from the Hs578T and MDA-MB-231 cell lines. The lentivirus-based expression vector pLKO10 (National RNAi Core Facility, Academia. Sinica, Taipei, Taiwan) with different miRNA was also used to examine the inhibitory effect of miR-139 on cancer cell invasion and migration.

### 4.5. Dual Luciferase Reporter Assay

pGL4.13 (Promega) was used to produce the recombinant vector pGL4.13/CXCR4 3′-UTR wild-type (WT). Briefly, pGL4.13 luciferase reporter constructs were prepared by cloning the binding sequence (WT/mutants) into the AgeI and EcoRI restriction enzyme sites complementary to the PCR amplified product of the *CXCR4* 3′-UTR. This cDNA fragment contains two miR-139 binding sites complementary to the *ACUGUAGA* sequence in the *CXCR4* 3′-UTR (Figure 2). *CXCR4* 3′-UTR/Mut1 and 3′-UTR/Mut2 (Figure 2) were obtained using a QuickChange II XL site-directed mutagenesis kit (Stratagene, La Jolla, CA, USA). Next, Hs578T and MDA-MB-231 cells were co-transfected with the WT or mutated 3′-UTR of *CXCR4* reporter construct and pLemiR containing miR-139 or pLemiR as the control group (Addgene, Cambridge, MA, USA), respectively. The relative ratio between firefly luciferase/Renilla luciferase activity was measured at 48 h after transfection by using the dual luciferase reporter assay system (Promega).

### 4.6. Wound Healing Assay and Matrigel Invasion Assay

A wound healing assay was performed to determine the migratory behavior of breast cancer cells [54]. Briefly, the cells were treated with plasmid carrying different miRNA fragments or anti-miR-mimic (NC), after which a sterile 10-μL tip was used to scratch the monolayer of cells to form a straight gap. The plate was incubated in a 37 °C, 5% CO_2_ incubator overnight. Images of a representative field of the cell-free space were acquired under a microscope at 0 h and 16 h after creating a scratch, and the distance of cell migration was calculated with Image Pro plus software (Media Cybernetics, Rockville, MD, USA). Besides, cell samples (5 × 10^4^ cells) were seeded into 48-well modified Boyden chambers (Neuro Probe, Cabin John, MD, USA) with 8-μm pore size polycarbonate membrane filters containing Matrigel for 16 h, and invading cells that had attached to the lower surface of the membrane were fixed with methanol and stained with Giemsa solution (Sigma). Invading cells were quantified by counting five random high-power fields using an Olympus Ckx41 light microscope (Tokyo, Japan).

### 4.7. Western Blotting Analysis

Expression levels of proteins in breast cancer cells were detected by western blot analysis as described previously [26]. Primary antibodies against human CXCR4 (GTX10403) and CXCR7 (GTX100027) (GeneTex, Irvine, CA, USA), AKT (sc-8312), ERK (sc-94), VEGF (sc-53462) and α-tubulin (sc-8035) (Santa Cruz Biotechnology, Dallas, TX, USA), phosphorylated AKT (1240003) (Calbiochem, San Diego, CA, USA), and IL-6 (12912S) and phosphorylated ERK (T202Y204) (Cell Signal Technology, Danvers, MA, USA) were used to validate the specificity of the proteins of interest. An antibody against α-tubulin was used as the endogenous control to normalize the expression of the proteins of interest. The immunoreactive protein band was visualized in an enhanced chemiluminescence assay (Western Blotting Luminol Reagent; Santa Cruz Biotechnology). Band intensities were quantified by densitometry (Digital Protein DNA Imagineware, Huntington Station, NY, USA).

### 4.8. Mouse Xenotransplantation Assay

All mice were housed in the animal facility at the Chung Shan Medical University Experimental Animal Center (Taichung, Taiwan). Approval was obtained from the Institutional Animal Care and Use Committee of Chung Shan Medical University (IACUC. No. 1278) for the use of animals, and all experiments were performed in accordance with the guidelines for animal care committee. For the orthotopic implantation model, 6–8-week-old female BALB/c nude mice were used. The breast cancer lung metastasis model was established by tail-vein injection of MDA-MB-231 cells (5 × 10^5^ cells in 0.1 mL of PBS). After 5 weeks, the mice were sacrificed by CO_2_ asphyxiation. The number of metastatic lung tumors was confirmed by HE staining under a dissecting microscope.

### 4.9. Patients and Tissue Samples

All current research procedures, including research design, sampling scheme, and consent procedure, were performed according to the National Research Council’s guide and were approved by the Ethics Committee of the Institutional Review Board at the Chung Shan Medical University Hospital (CSMUH No: CS12225). Frozen tissue specimens were histologically confirmed to be primary breast invasive ductal carcinoma (IDC) based on their pathological features, which were reviewed by two pathologists independently. A total of 191 pairs of cancer and non-tumor cells were collected from patients with breast cancer enrolled between 1999 and 2006. All participants provided written informed consent to participate in this study. No patients underwent radiotherapy or chemotherapy before surgery. The clinicopathological characteristics of the cohort are summarized in Table 3.

### 4.10. Laser Capture Microdissection and Quantitative Real-Time PCR

The detailed procedure for RNA isolation from cells collected by the LCM technique has been described elsewhere [26]. RNA was extracted from LCM–collected samples of the tumor and neighboring non-tumor breast cells of each patient using a mirVana miRNA isolation kit (Ambion, Austin, TX, USA). The RNA concentration was estimated with a NanoDrop 1000 spectrophotometer (NanoDrop Technologies, Wilmington, DE, USA). An appropriate probe and primer set was used to detect the expression of genes encoding hsa-miR-139 (AB assay ID: 17100) and then subjected to the single-tube TaqMan miRNA assay (Applied Biosystems, Foster City, CA, USA) on an Applied Biosystems instrument. The results were normalized against *RNU6B*. Fold-changes in expression were calculated by relative quantification using the 2^−ΔΔCT^ method with three independent experiment.

### 4.11. Statistical Analysis

Data are presented as the mean ± standard deviation (S.D.) of three independent replicates in experiments using different cell lines and in mice model experiments. The statistical significance of the experimental data grouped by one variable was evaluated by unpaired Student’s *t*-test, Mann–Whitney U test for two-group comparisons, one-way analysis of variance with Tukey’s multiple comparison test, or Kruskal-Wallis test for three-group comparison where appropriate. The clinicopathological characteristics of patients with breast cancer are presented as the mean ± S.D. for continuous variables or as the proportion for categorical data. The associations of miR-139 expression with different clinicopathological characteristics of patients were evaluated by chi-square or Fisher’s exact tests, and odds ratios (ORs) and 95% confidence intervals (CIs) were determined by logistic regression. All tests were two-sided, and differences with *p* < 0.05 were considered statistically significant. All statistical analyses were performed using SPSS version 19.0 for Windows (SPSS, Inc., Chicago, IL, USA).

## 5. Conclusions

Our data showed that miR-139 inhibits breast cancer cell stemness by directly targeting CXCR4 at the molecular and cellular levels as well as the results from in vivo mouse xenotransplantation assay. The proliferative and progression advantages of BCSCs that are enhanced via CXCR4-mediated activation of p-Akt signaling was attributed to miR-139 suppression by the addition of miR-139 antagomiR. Oppositely, overexpression of miR-139 was shown to correlate with reduced invasion and migration abilities in breast cancer. Thus, the oncogenic advantage and aggressive progression of BCSCs are enhanced by activation of CXCR4/p-Akt signaling pathway due to loss of miR-139. This indicates its potential as a promising therapeutic target that may help design better strategies to treat breast cancer patients.

## Figures and Tables

**Table 1:** Clinicopathological significance of miR-139 in patients with IDC.

Clincopathological Parameters	Decreased miR-139 Levels (T/N Ratio, −ddct < −2.20)	
	*N* (%)	OR (95% CI)
Tumor size (mm^3^)		
≤20	48/106 (45.3)	1.00 (Ref.)
>20	51/85 (60.0)	1.81 (1.02–3.23) *
Tumor grade		
Well (G1)	10/24 (41.7)	1.00 (Ref)
Moderate (G2)	39/82 (47.6)	1.27 (0.51–3.19)
Poor (G3)	50/85 (58.8)	2.01 (0.79–5.02)
Tumor stage		
I	29/65 (44.6)	1.00 (Ref)
IIa/IIb	48/96 (50.0)	1.24 (0.66–2.34)
III	18/26 (69.2)	2.79 (1.06–7.34) *
IV	4/4 (100.0)	--
LNM		
N_0_	45/102 (44.1)	1.00 (Ref)
N_1_	35/60 (58.3)	1.77 (0.93–3.38)
N_2_	19/29 (65.5)	2.41 (1.02–5.69) *

Cutoff value for low miR-139 levels was set at four-fold decrease (2^−2.2^ = 0.22) in micro-dissected tumor cells compared non-tumor cells; OR (95% CI), logistic regression model with odds ratio and 95% confidence interval; Lymph node status was classified as N_0_ (LNM-negative) and N_1_, and N_2_ (LNM-positive); Ref, reference group; * *p* < 0.05.

**Table 2:** Associations between decreased expression of miR-139 and clinicopathological features of breast cancer stratified by ER, PR, or HER2 statuses.

	Decreased miR-139 Expression in Cancer Tissue Relative to Non-Cancer Tissues (T/N Ratio, 2^−2.2^ = 0.22)											
	ER				PR				HER2			
	Positive		Negative		Positive		Negative		Negative		Positive	
	N (%)	OR (95% CI)	N (%)	OR (95% CI)	N (%)	OR (95% CI)	N (%)	OR (95% CI)	N (%)	OR (95% CI)	N (%)	OR (95% CI)
Tumor size (mm^3^)												
≤20	25/55 (45.5)	1.00 (Ref.)	23/50 (46.0)	1.00 (Ref.)	28/53 (52.8)	1.00 (Ref.)	20/51 (39.2)	1.00 (Ref.)	30/65 (46.2)	1.00 (Ref.)	18/40 (45.0)	1.00 (Ref.)
>20	13/26 (50.0)	1.20 (0.47–3.05)	36/57 (63.2)	2.01 (0.92–4.36)	18/33 (54.5)	1.07 (0.45–2.56)	31/50 (62.0)	2.53 (1.13–5.64) *	24/45 (53.3)	1.33 (0.62–2.86)	25/38 (65.8)	2.35 (0.94–5.87)
Grade												
I/II	27/59 (45.8)	1.00 (Ref.)	21/45 (46.7)	1.00 (Ref.)	27/55 (49.1)	1.00 (Ref.)	21/48 (43.8)	1.00 (Ref.)	27/62 (43.5)	1.00 (Ref.)	21/42 (50.0)	1.00 (Ref.)
III	11/22 (50.0)	1.19 (0.45–3.16)	38/62 (61.3)	1.81 (0.83–3.94)	19/31 (61.3)	1.64 (0.67–4.02)	30/53 (56.6)	1.68 (0.76–3.69)	27/48 (56.3)	1.67 (0.78–3.56)	22/36 (61.1)	1.57 (0.64–3.88)
Stage												
I/IIa/IIb	34/74 (45.9)	1.00 (Ref.)	41/84 (48.8)	1.00 (Ref.)	38/75 (50.7)	1.00 (Ref.)	37/82 (45.1)	1.00 (Ref.)	45/96 (46.9)	1.00 (Ref.)	30/62 (48.4)	1.00 (Ref.)
Ⅲ/Ⅳ	4/7 (57.1)	1.57 (0.33–7.50)	18/23 (78.3)	3.78 (1.28–11.11) *	8/11 (72.7)	2.60 (0.64–10.55)	14/19 (73.7)	3.41 (1.12–10.33) *	9/14 (64.3)	2.04 (0.64–6.54)	13/16 (81.3)	4.62 (1.20–17.84) *
LNM												
Negative	20/44 (45.5)	1.00 (Ref.)	25/57 (43.9)	1.00 (Ref.)	22/44 (50.0)	1.00 (Ref.)	23/57 (40.4)	1.00 (Ref.)	29/65 (44.6)	1.00 (Ref.)	16/36 (44.4)	1.00 (Ref.)
Positive	18/37 (48.6)	1.14 (0.47–2.73)	34/50 (68.0)	2.72 (1.23–6.00) *	24/42 (57.1)	1.33 (0.57–3.12)	28/44 (63.6)	2.59 (1.15–5.82) *	25/45 (55.6)	1.55 (0.72–3.33)	27/42 (64.3)	2.25 (0.90–5.59)

LNM, lymph node metastasis; ER, estrogen receptor; PR, progesterone receptor; HER2, human epidermal growth factor receptor 2; N/A, data not available. Tumor classification was referred to eighth edition of the American Joint of Committee on Cancer (AJCC) Cancer Staging Manual (2017). ER, PR or HER2 status was classed as negative when fewer than 30% of cells were stained and as positive with more than 30% staining. Ref, reference group. * *p* < 0.05.

**Table 3:** Clinicopathological characteristics of IDC patients (*N* = 191).

Clinicopathological Characteristics	N (%)
Age (mean ± S.D. and range, years)	50.8 ± 11.7 (23–87)
Tumor size (mm^3^)	
≤20	106 (55.5)
>20	85 (44.5)
Histological grade	
Well differentiation (G1)	24 (12.6)
Moderate differentiation (G2)	82 (42.9)
Poor differentiation (G3)	85 (44.5)
Clinical stage	
I	65 (34.0)
IIa	70 (36.7)
IIb	26 (13.6)
III	26 (13.6)
IV	4 (2.1)
LNM	
Negative (N_0_)	102 (53.4)
Positive (N_1_/N_2_)	89 (46.6)
ER	
Positive (ER+)	81 (43.1)
Negative (ER−)	107 (56.9)
N/A	3
PR	
Positive (PR+)	86 (46.0)
Negative (PR−)	101 (54.0)
N/A	4
HER2/neu	
Negative (HER2−)	110 (58.5)
Positive (HER2+)	78 (41.5)
N/A	3
Molecular subtype	
Luminal A	67 (35.8)
Luminal B	38 (20.3)
HER2-enriched	40 (21.4)
TNBC	42 (22.5)
N/A	4

Luminal A (ER+ and/or PR+, and HER2−), Luminal B (ER+ and/or PR+ and HER2+), HER2-enriched (ER−, PR−, and HER2+), and TNBC, triple-negative breast cancer (ER−, PR−, and HER2−).

## Data Availability

All data presented in this study are available on request from the corresponding author.

## Supplementary Materials

The following are available online at https://www.mdpi.com/article/10.3390/cancers13112582/s1 Figure S1: Increased miR-139 levels did not affect cell cycle distribution of BCSCs. Figure S2. Expression levels of miR-139 in molecular subtypes of breast cancer. Figure S3. Association between clinical outcome and hsa-miR-139-5p in breast cancer patients. Table S1: DNA sequence of primer set for biosynthesis of the primary-miRNA transcript. Table S2. Association between clinicopathological features and decreased expression of miR-139 with respect of the breast cancer subtypes. File S1: Original Images for Blots.

## Abbreviations

miRNA	MicroRNA
CXCR4	Chemokine receptor 4
CSC	Cancer stem cell
3′-UTR	3′-untrnasltaed region
HER2/neu	Human epidermal growth factor receptor 2
Akt	Protein kinase B
EMT	Epithelial mesenchymal transition
FACS	Fluorescence-activated cell sorting
IDC	Invasive ductal carcinoma
LNM	Lymph node metastasis
LCM	Laser capture microdissection
qRT-PCR	Quantitative real-time PCR
