# Supplementary material for: MiR-139 Modulates Cancer Stem Cell Function of Human Breast Cancer through Targeting CXCR4
Source: Cancers (Basel). 2021 May 25;13(11):2582. doi: 10.3390/cancers13112582 (PMC8198393; doi:10.3390/cancers13112582)
Supplement: Supplementary file 1 [file cancers-13-02582-s001.zip › XML-suppl/Original Images for Blots.pdf]

Western blots of Figure 2E

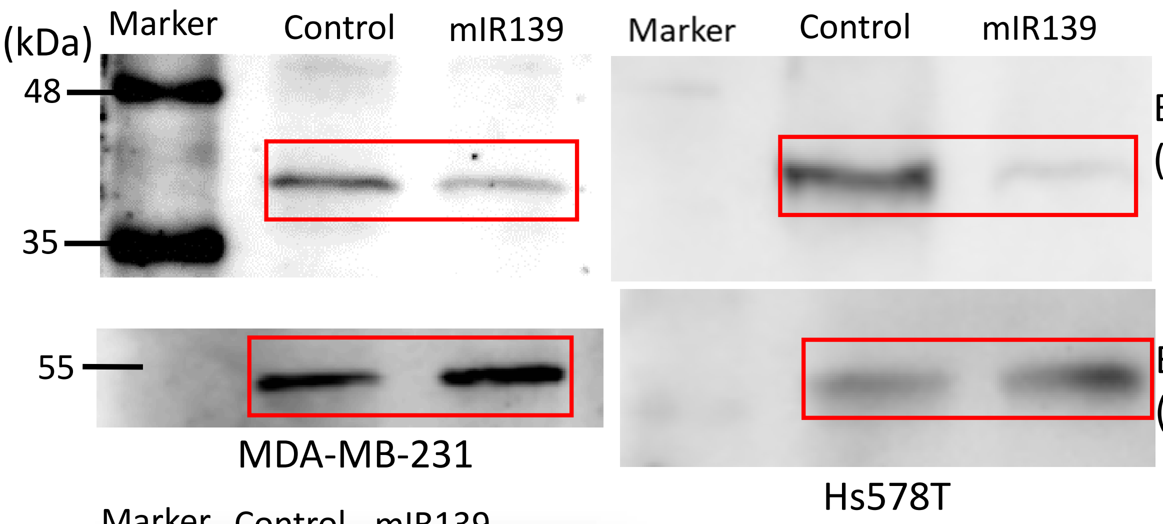

Blots probed with anti-CXCR4 (GeneTex GTX10403; 40 kDa)

Blots probed with anti-α-tubulin (Santa Cruz SC-8035; 55 kDa)

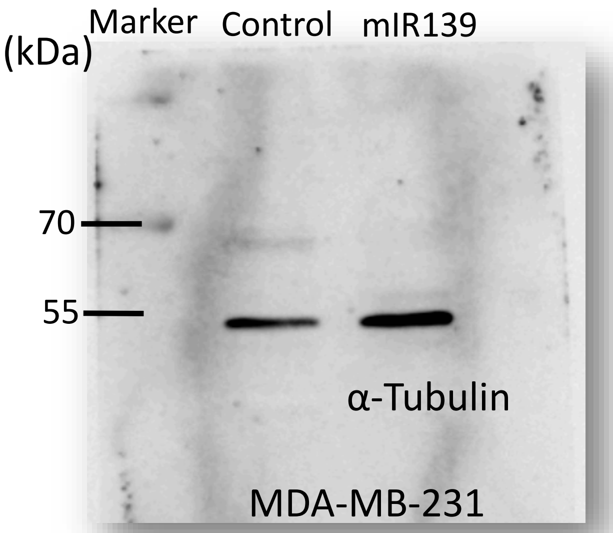

Western blots of Figure 3C

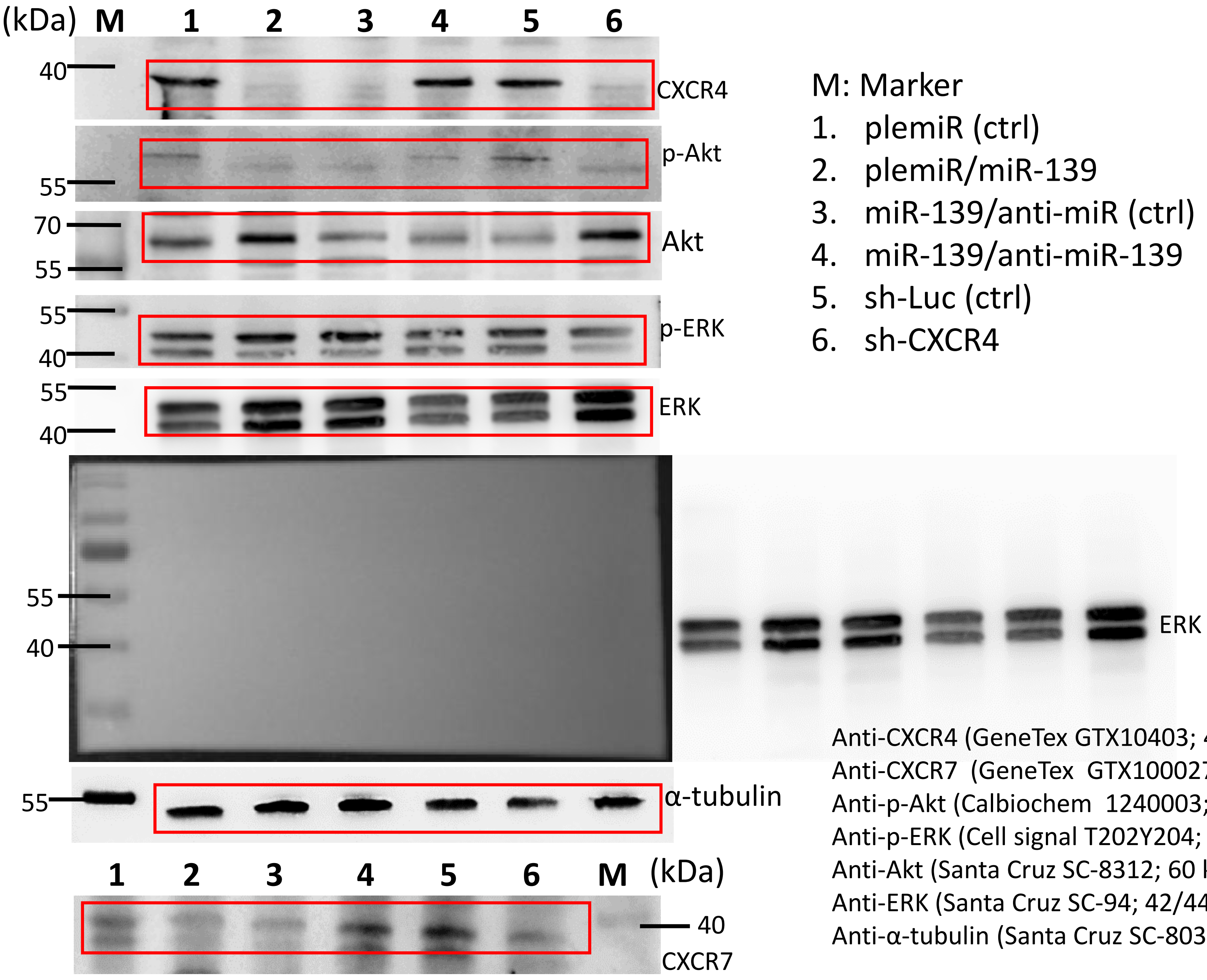

M: Marker

1. plemiR (ctrl)
2. plemiR/miR-139
3. miR-139/anti-miR (ctrl)
4. miR-139/anti-miR-139
5. sh-Luc (ctrl)
6. sh-CXCR4

Anti-CXCR4 (GeneTex GTX10403; 40 kDa)
Anti-CXCR7 (GeneTex GTX100027; 41 kDa)
Anti-p-Akt (Calbiochem 1240003; 60 kDa)
Anti-p-ERK (Cell signal T202Y204; 42/44 kDa)
Anti-Akt (Santa Cruz SC-8312; 60 kDa)
Anti-ERK (Santa Cruz SC-94; 42/44 kDa)
Anti-α-tubulin (Santa Cruz SC-8035; 55 kDa)

Western blots of Figure 4F

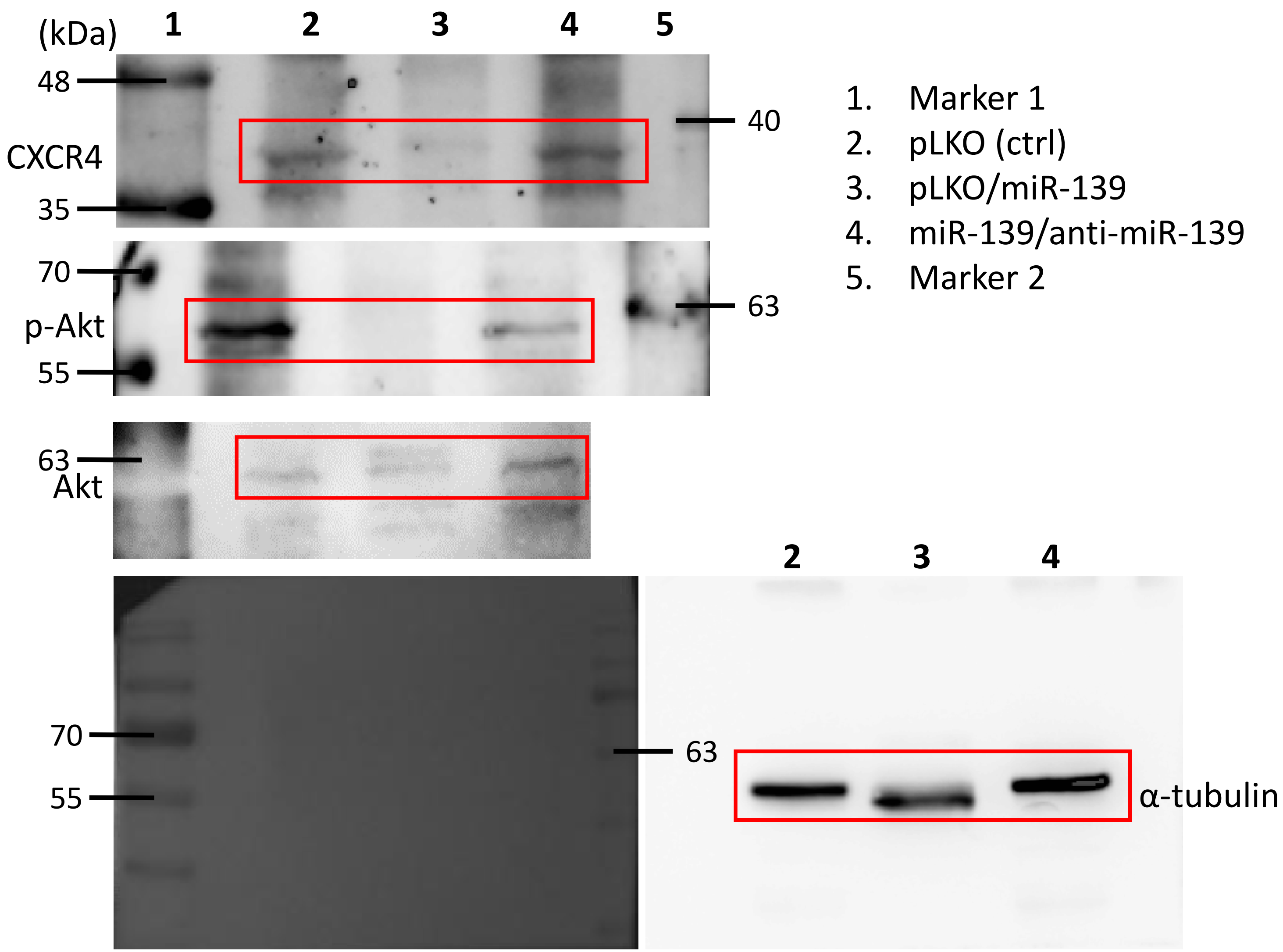

1. Marker 1
2. pLKO (ctrl)
3. pLKO/miR-139
4. miR-139/anti-miR-139
5. Marker 2

Anti-CXCR4 GeneTex GTX10403; 40 kDa
Anti-p-Akt Calbiochem 1240003; 60 kDa
Anti-Akt Santa Cruz SC-8312; 60 kDa
Anti-α-tubulin Santa Cruz SC-8035; 55 kDa

Western blots of Figure 5C

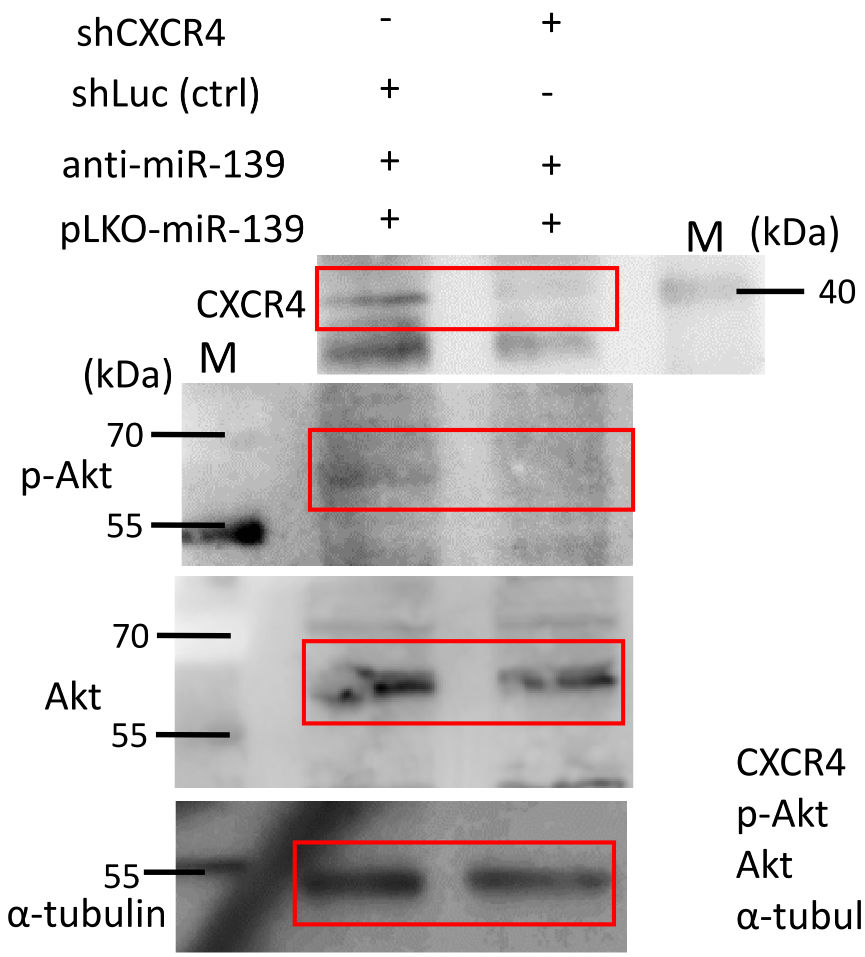

CXCR4 GeneTex GTX10403; 40 kDa
p-Akt Calbiochem 1240003; 60 kDa
Akt Santa Cruz SC-8312; 60 kDa
α-tubulin Santa Cruz SC-8035; 55 kDa

Western blots of Figure 5G

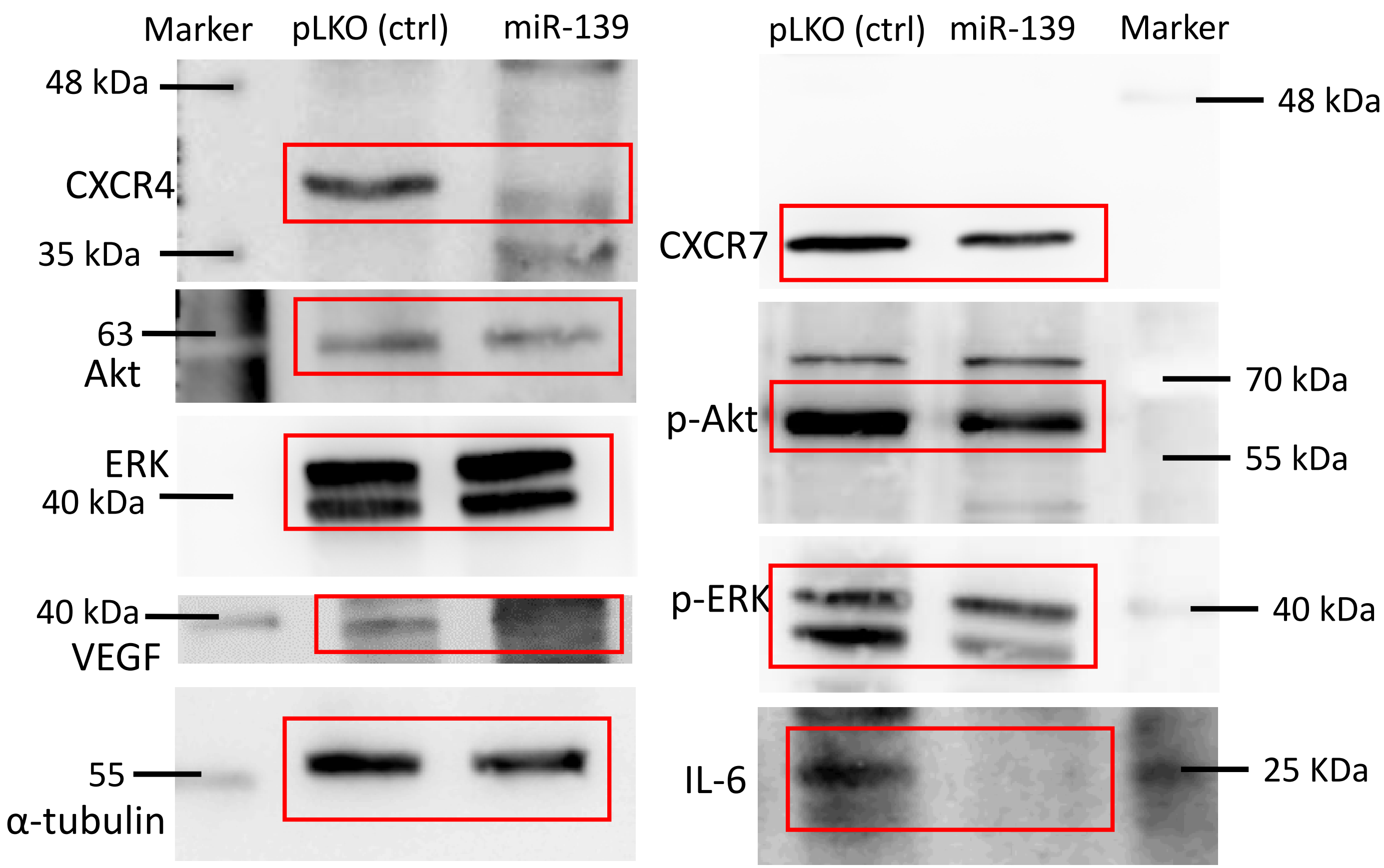

CXCR4 GeneTex GTX10403; 40 kDa
CXCR7 GeneTex GTX100027; 41 kDa
p-Akt Calbiochem 1240003; 60kDa
Akt Santa Cruz SC-8312; 60kDa
p-ERK Cell signal T202Y204; 42/44 kDa
IL-6 Cell signal 12912S; 24 kDa
ERK Santa Cruz SC-94; 42/44 kDa
VEGF Santa Cruz SC-53462; 40 kDa
α-tubulin Santa Cruz SC-8035; 55 kDa
