# Supplementary material for: MiR-139 Modulates Cancer Stem Cell Function of Human Breast Cancer through Targeting CXCR4
Source: Cancers (Basel). 2021 May 25;13(11):2582. doi: 10.3390/cancers13112582 (PMC8198393; doi:10.3390/cancers13112582)
Supplement: Supplementary file 1 [file cancers-13-02582-s001.zip › XML-suppl/Supplementary Materials.pdf]

Supplementary Files.

Table S1. DNA sequence of primer set for biosynthesis of the primary-miRNA transcript

Name	Nucleotide sequence of the primer
hsa-miR-34a	Forward : 5'- GTGACCGGTAGGCACGCATGTCACCAT-3'
	Reverse : 5'- CCCGGAATTCCAAACTTCTCCCAGCCAA-3'
hsa-miR-135a	Forward : 5'- GTGACCGGTAGGTGGTGGAAGATGGTGA -3'
	Reverse : 5'- CCGGAATTCCGATCCCAGGTTACCAGATC -3'
hsa-miR-139	Forward : 5'- GTGACCGGTGAGGCAGGAGCTGGAATAGA -3'
	Reverse : 5'- CCGGAATTCACCTGCCAGAGACCTTTCCTC -3'
hsa-miR-519e	Forward : 5'- TCCACCGGTAGGAACTGGAGATGGT-3'
	Reverse : 5'- CCCGGAATTCTGTGGTGAAACTCCAT-3'
hsa-miR-217	Forward : 5'- GTGACCGGTGCAACACAGCAAGACTCCA -3'
	Reverse : 5'- CCGGAATTCGACCATGAACACATCACAGC -3'
hsa-miR-218-1	Forward : 5'- GTGACCGGTGTGGAGGCACCTTTTCCATA -3'
	Reverse : 5'- CCCGGAATTCGGGCTTTCAAGGCAAATAGA -3'
hsa-miR-95	Forward : 5'- TCCACCGGTTCTCGGGGTTCTTTTGATTG-3'
	Reverse : 5'- CGGAATTCGTATCTGGTGGAGGGATGGA-3'
hsa-miR-329	Forward : 5'- GTGACCGGTTTAACCACGAAGCCTGTG -3'
	Reverse : 5'- CCCGGAATTCACATTGAGGGTTAGCGCAAT -3'
hsa-miR-345	Forward : 5'- GTGACCGGT CGTTTCCAGGTTTAGGGTCA -3'
	Reverse : 5'- CCCGGAATTCATGGATGTGCAGGGATTTGT -3'
hsa-miR-489	Forward : 5'- GTGACCGGTATGATGTTTGGGCTGCTGA -3'
	Reverse : 5'- CCGGAATTCGAATTGCTGGGAAGGTGGT -3'
hsa-miR-491	Forward : 5'- GTGACCGGTAAAGATGGCTTTCTGGGT -3'
	Reverse : 5'- CCCGGAATTCAAATAGCCATCCTACAC -3'

hsa-miR-10a	Forward : 5'- TCCACCGGTACAGACTCGCACTGCCTTTT -3'
	Reverse : 5'- CGGAATTCCGTGGGGAGAGTTCAGGTAG -3'
hsa-miR-197	Forward : 5'- TTCACCGGTTGCAGTCTCCAGTGTCTTCTG-3'
	Reverse : 5'- CCCCGGAATTCAGCGGCACAAAAGTTGTCTT-3'
hsa-miR-380	Forward : 5'- TCCACCGGTAACGCATGTTACTTCGTCCA-3'
	Reverse : 5'- CGGAATTCAGGGCTGGCTGATACTGAGA -3'
hsa-miR-501	Forward : 5'- TCCACCGGTTGCTAGGCCCCAAATACATC-3'
	Reverse : 5'- CCCGGAATTCGCGAAGTCTGCCTTGTTCAT-3'
hsa-miR-502	Forward : 5'- TCCACCGGTCACAACATGGGACTTC-3'
	Reverse : 5'- CCCGGAATTCGCTCCATCTCATTGAA-3'
hsa-miR-572	Forward : 5'- TTCACCGGTGAAGCCGGTCCACTCCTC-3'
	Reverse : 5'- CCCGGAATTCGTCCGCGTTCACTCACAACT-3'

A

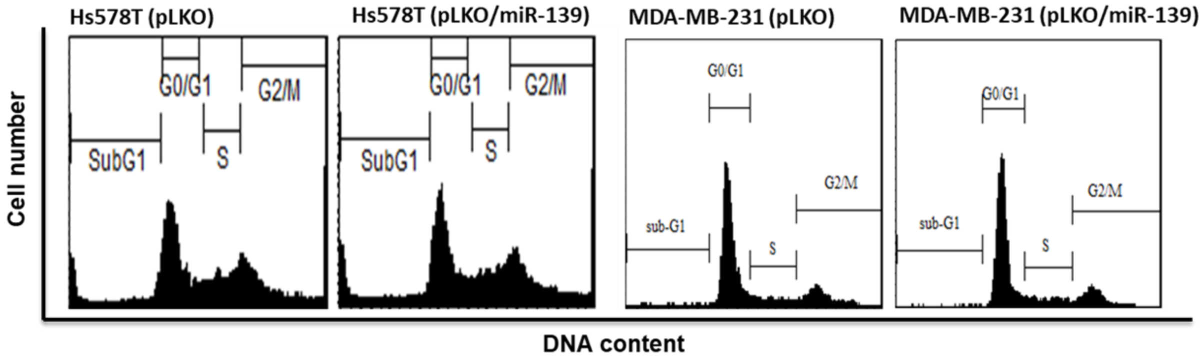

B

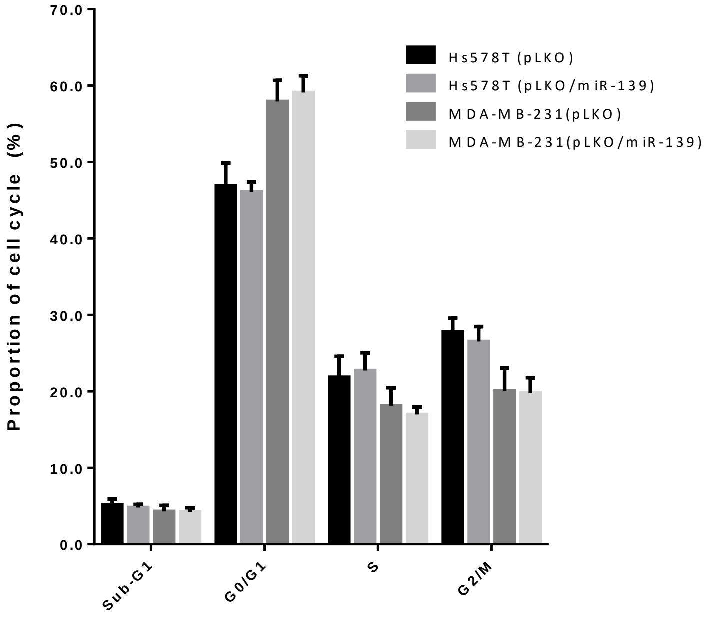

Figure S1. Increased miR-139 levels did not affect cell cycle distribution of BCSCs. (**A**) Schematic representation of the distribution of MDA-MB-231 SPs across each phase of the cell cycle as determined by flow cytometric analysis. (B) SPs derived from Hs578T cells and MDA-MB-231 cell lines were cultured for 48 h. Percentage of cells at different stages of the cell cycle are shown as means ± SD of three independent experiments.

.

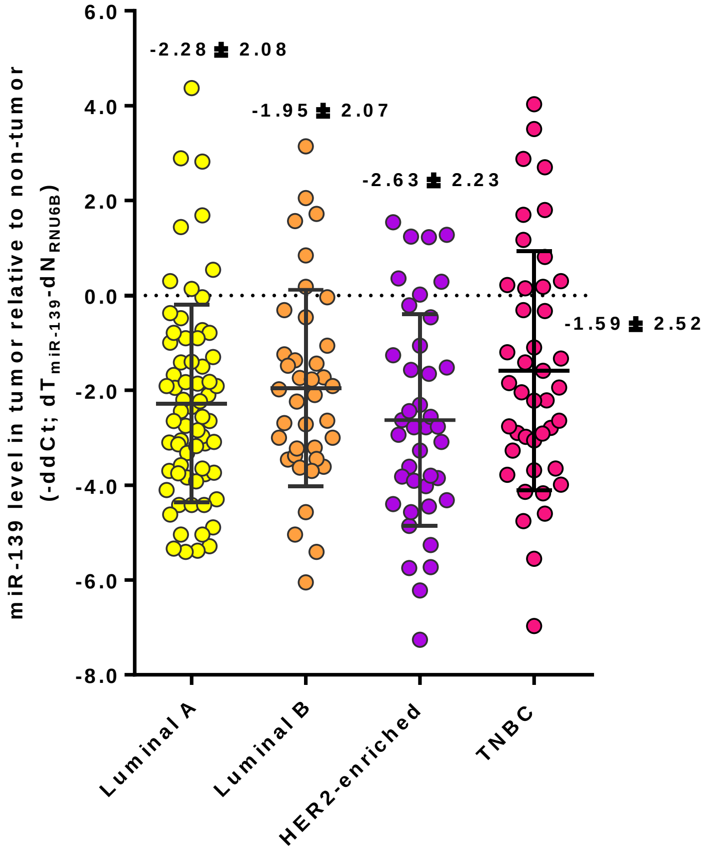

Figure S2. Expression levels of miR-139 in molecular subtypes of breast cancer. Expression level of miR-139 from LCM-captured cancerous and adjacent noncancerous cells was measured by qRT-PCR. Each dot represents a case, the middle line marks the mean, and the upper and lower depict the borders of the 25 and 75 % quartiles

Table S2. Association between clinicopathological features and decreased expression of miR-139 with respect of the breast cancer subtypes

	Decreased miR-139 expression in cancer tissue relative to non-cancer tissues (T/N ratio, $2^{-2.2} = 0.22$)							
	Luminal A (N=67)		Luminal B (N=38)		HER2-enriched (N=40)		TNBC (N=42)	
Variable	N (%)	OR (95% CI)	N (%)	OR (95% CI)	N (%)	OR (95% CI)	N (%)	OR (95% CI)
Tumor size (mm^3)								
≦20	23/43 (53.5)	1.00 (Ref.)	9/23 (39.1)	1.00 (Ref.)	9/17 (52.9)	1.00 (Ref.)	7/21 (33.3)	1.00 (Ref.)
＞20	13/24 (54.2)	1.03 (0.38-2.80)	8/15 (53.3)	1.78 (0.48-5.62)	17/23 (73.9)	2.52 (0.67-9.53)	11/21 (52.4)	2.20 (0.63-7.66)
Grade								
I/II	21/42 (50.0)	1.00 (Ref.)	10/25 (40.0)	1.00 (Ref.)	11/18 (61.1)	1.00 (Ref.)	6/18 (33.3)	1.00 (Ref.)
III	15/25 (60.0)	1.50 (0.55-4.09)	7/13 (53.8)	1.75 (0.45-6.77)	15/22 (68.2)	1.19 (0.45-3.16)	12/24 (50.0)	2.00 (0.56-7.09)
Stage								
I/IIa/IIb	32/59 (54.2)	1.00 (Ref.)	12/31 (38.7)	1.00 (Ref.)	19/31 (61.3)	1.00 (Ref.)	13/36 (36.1)	1.00 (Ref.)
III/IV	4/8 (50.0)	0.84 (0.19-3.70)	5/7 (71.4)	3.96 (0.67-23.76)	7/9 (77.8)	2.21 (0.39-12.47)	5/6 (83.3)	8.85 (0.93-8.41)
LNM								
Negative	19/36 (52.8)	1.00 (Ref.)	6/17 (35.3)	1.00 (Ref.)	10/19 (45.5)	1.00 (Ref.)	10/29 (34.5)	1.00 (Ref.)
Positive	17/31 (54.8)	1.09 (0.42-2.85)	11/21 (52.4)	2.02 (0.54-7.49)	16/21 (48.6)	2.88 (0.75-11.10)	8/13 (61.5)	3.04 (0.79-11.78)

Ref, reference group.

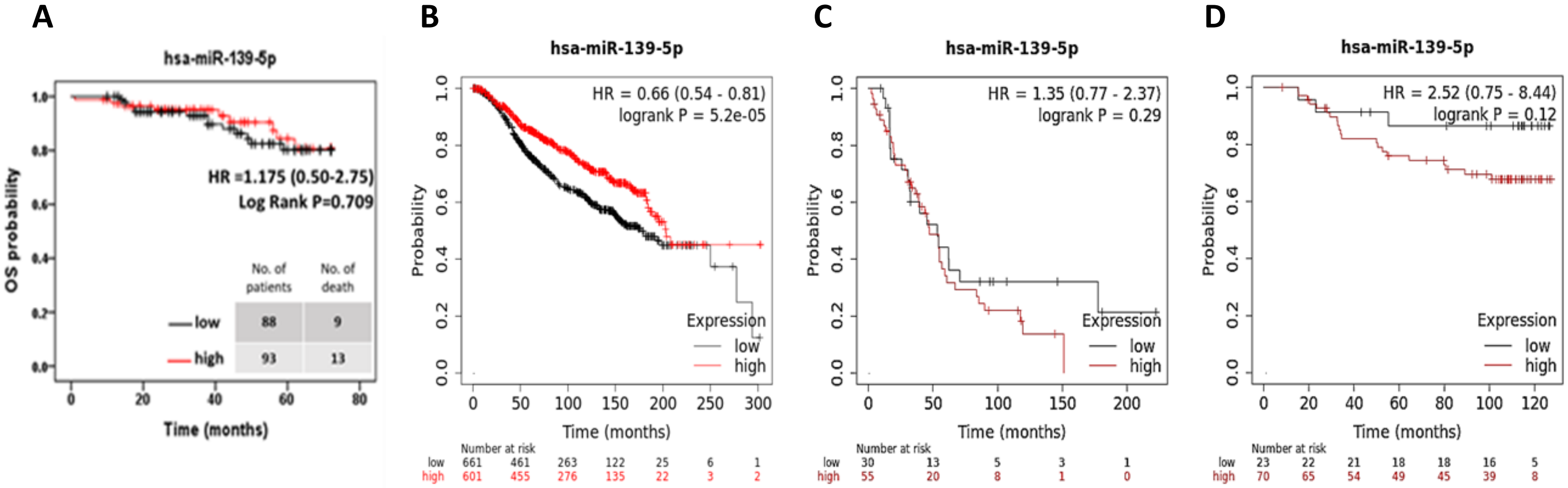

Figure S3. Association between clinical outcome and hsa-miR-139-5p in breast cancer patients. Kaplan–Meier statistical analyses examining the association between OS and low level of miR-139-5p in studies from the database of our breast cancer cohort (*N*=181) in (A), METABRIC (Molecular Taxonomy of Breast Cancer International Consortium, *N* = 1262) in (B), and two independent cohorts of GSE40267 (*N* = 173) and GSE19783 (*N* = 101) in (C) and in (D), respectively
